# Affective responses in mountain hiking—A randomized crossover trial focusing on differences between indoor and outdoor activity

**DOI:** 10.1371/journal.pone.0177719

**Published:** 2017-05-16

**Authors:** Martin Niedermeier, Jürgen Einwanger, Arnulf Hartl, Martin Kopp

**Affiliations:** 1 Department of Sport Science, University of Innsbruck, Innsbruck, Austria; 2 Austrian Alpine Association, Innsbruck, Austria; 3 Institute of Ecomedicine, Paracelsus Medical University, Salzburg, Austria; IRCCS E. Medea, ITALY

## Abstract

**Introduction:**

Affective responses during physical activity (PA) are important for engagement in PA programs and for adherence to a physically active lifestyle. Little is known about the affective responses to PA bouts lasting longer than 45 minutes. Therefore, the aims of the present study were to analyse acute effects on affective responses of a three-hour outdoor PA intervention (mountain hiking) compared to a sedentary control situation and to an indoor treadmill condition.

**Methods:**

Using a randomized crossover design, 42 healthy participants were randomly exposed to three different conditions: outdoor mountain hiking, indoor treadmill walking, and sedentary control situation (approximately three hours each). Measures included the Feeling Scale, Felt Arousal Scale and a Mood Survey Scale. Repeated measures ANOVAs were used to analyse differences between the conditions.

**Results:**

Compared to the control situation, the participants showed a significant increase in affective valence (*d* = 1.21, *p* < .001), activation (*d* = 0.81, *p* = .004), elation (*d* = 1.07, *p* < .001), and calmness (*d* = 0.84, *p* = .004), and a significant decrease in fatigue (*d* = -1.19, *p* < .001) and anxiety (*d* = -.79, *p* < .001) after mountain hiking. Outdoor mountain hiking showed significantly greater positive effects on affective valence, activation, and fatigue compared to indoor treadmill walking.

**Discussion:**

The results indicate that a three-hour PA intervention (mountain hiking) elicits higher positive and lower negative affective responses compared to a sedentary control situation and to an indoor PA condition. Outdoor mountain hiking can be recommended by health professionals as a form of PA with the potential to positively influence affective responses.

**Trial registration:**

ClinicalTrials.gov NCT02853760. https://clinicaltrials.gov/. Date of registration: 08/02/2016 (retrospectively registered). Date of enrolment of the first participant to the trial: 05/01/2014.

## Introduction

Physical activity (PA) is believed to serve as a panacea for different diseases and may also prevent premature mortality [1, 2]. Nevertheless, more than 70% of the European population show insufficient health-enhancing physical activity rates [3]. Therefore, a large body of literature focused on the mechanisms behind increasing the rate of physically active people [4]. Among other strategies, there are efforts to increase inactive people’s motivation to start a PA program [5–8] and already active people to maintain their PA behaviour [9, 10]. At both stages of starting and maintaining a PA program, affective responses to PA seem to play an important role. In accordance to Ekkekakis & Petruzzello [11], affective responses to PA are considered as the products of the continuous interplay between “interoceptive cues about the physiological condition of the body” and cognitions, “such as considerations related to self-efficacy, selfpresentational concerns, goals, or attributions” [11]. Basic dimensions of affect are used to operationalize affective responses, e.g. affective valence and perceived activation. Affective valence can be considered as the degree of pleasure (or displeasure) in a specific situation, while perceived activation is used for the feeling of arousal (high or low) [12]. Several studies have shown a positive relationship between positive affective responses during PA and future PA [9, 13, 14]. This relationship may also exist when only a single bout of PA is investigated [9]. Based on this knowledge, the relationship between affective responses and PA needs to be addressed in a comprehensive and health-related dimension.

Many studies reported positive effects of PA on affective responses in people with various health statuses [12, 15–18]. However, different effects on affective responses have been shown depending on the intensity of PA [19, 20]. Ekkekakis [21] addressed this phenomenon in his dual-mode theory. Although intensity is no doubt an important characteristic of PA, there are further parameters to consider, e.g. PA duration. Studies on the effects of a single bout of PA typically were undertaken with durations of 15 to 30 minutes [19]. Although some studies compared different PA bouts up to 45 minutes and proposed a U-relationship between PA duration and affective responses [22], little is known about the effects of PA with a longer duration.

Mountain hiking, considered as walking in mountainous areas with altitude differences, seems to be a form of PA with a longer duration. Despite the PA duration varies a lot among mountain hikers, tours with a mean duration of two to three hours were reported previously [23, 24]. Therefore, mountain hiking can be considered as an appropriate example to study effects of longer-lasting physical activity interventions.

Studying the effects of mountain hiking on affective responses may also offer new insights in environmental aspects of PA. The importance of environmental effects on PA behaviour has been recognized. Sallis & Cerin [25] emphasized the importance of the city design aiming on the enhancement of physical activity. However, changing the environment for increasing PA is a long-term project and it seems important to follow multidimensional approaches to change adherence to PA. Affective responses, and consequently adherence to PA, can also be influenced by the surrounding environment. Indeed, PA in a natural environment (green exercise) has been shown to create larger positive effects on affective responses compared to indoor PA [26–30]. In their meta-analysis, Barton & Pretty [28] provided evidence for a dose-response relationship between the duration of green exercise and the impact on affective responses. According to the authors, the largest effect on affective responses (i.e., mood measured by the Profile of Mood State) was shown after 5 minutes of green exercise and the smallest effect after half a day of green exercise. A limiting aspect is that those studies used a pre-post measurement without control condition or control group. Furthermore, most of the green exercise studies investigated a short bout of PA and we could not detect studies containing a longer duration of green exercise with a control group or control situation.

Therefore, the aims of the present study were (1) to analyse affective responses of a single bout of long-duration PA (mountain hiking, approximately three hours) and (2) to detect possible green exercise effects in a randomized crossover trial using an outdoor PA-condition, an indoor-PA-condition as well as a physically inactive condition of the same duration.

We hypothesized positive changes in affective responses due to a single bout of mountain hiking. Concordant with the literature on green exercise, we further hypothesized that outdoor mountain hiking will result in larger positive effects in affective responses compared to an indoor PA intervention.

## Materials and methods

### Design

The project was designed as a randomized crossover study, in which all participants were exposed to three experimental conditions in a randomized order: outdoor mountain hiking (M), indoor treadmill walking (T) and sedentary control condition (C). Comparing M and C enabled us to examine the intervention effect, while comparisons between M and T conditions could illustrate potential green exercise effects. A crossover design was used to reduce error variance associated with inter-individual differences and to increase statistical power. To control for psychosocial group effects, all assessments were done in groups (mean group size: five participants). The mean washout phase between the conditions was 1 week; however, due to conflicting schedules, the washout phase varied from one to 14 days.

Three independent study waves were conducted to be able to organise all measurements and participants. Wave one took place in May 2015, wave two in August 2015 and wave three in September/October 2015. Recruitment started two weeks before each wave. The daytime of the three study waves differed by one hour because to the sunset. Within one wave, all interventions were started at the same time of the day (2, 3, 4 p.m. respectively). All waves were conducted at the same places in Innsbruck, Austria.

The study was approved by the Institutional Review Board of the Department of Sport Science of the University of Innsbruck (22.04.2015) and all participants signed a consent form after obtaining written and spoken information about the study procedures. The study was registered retrospectively, since a healthy, non-clinical population was included and since trial registration was not considered as necessary for the interventions (walking) and measurements (mainly questionnaires). The authors confirm that all ongoing and related trials for this intervention are registered.

### Participants

The required sample size was based on an a priori power analysis with the following assumptions. The effect size was set to *d* = .54 based on previous studies [28, 30, 31]. Using G*Power 3.1 [32], we calculated a total sample size of 38 participants to detect effects with a power of .80 in the conditions by time interaction in a three × two repeated measures ANOVA. Assuming a dropout rate of 20%, the sample size was adjusted to 46 subjects.

**Fig 1:**
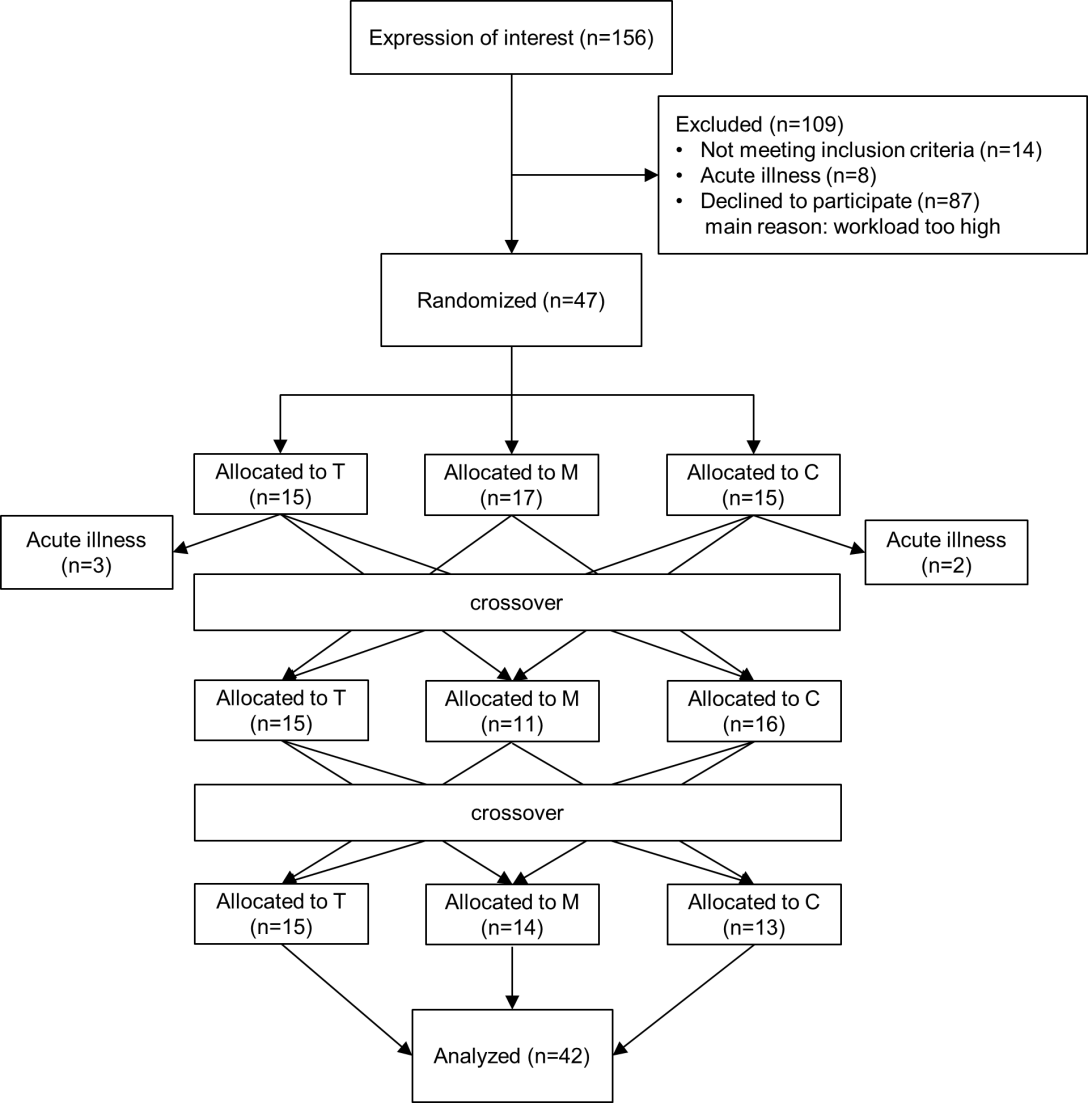
Participant flow diagram. M: outdoor mountain hiking, T: indoor treadmill walking, C: sedentary control condition.

The participants were recruited in two waves by public announcement on the web page of the Austrian Alpine Association and by e-mail announcements at the University of Innsbruck. A detailed flowchart of the participants is presented in Fig 1. From 156 interested participants, 14 did not meet the eligibility criteria. The exclusion criteria were: (1) pregnancy, (2) breast-feeding, (3) chronic or acute diseases (already existing or diagnosed during the study), (4) age below 18 and above 70 years, (5) unable to be physically active assessed by the Physical Activity Readiness Questionnaire [33]. No incentive was provided for the participation in the study. In total, 47 participants were randomized and 42 participants completed the study.

### Procedure

All participants were randomly exposed to three experimental conditions using simple randomisation method by MN. 10 study blocks were needed to test all the participants. In each block, there were three to eight participants (mean group size: five).

**Fig 2:**
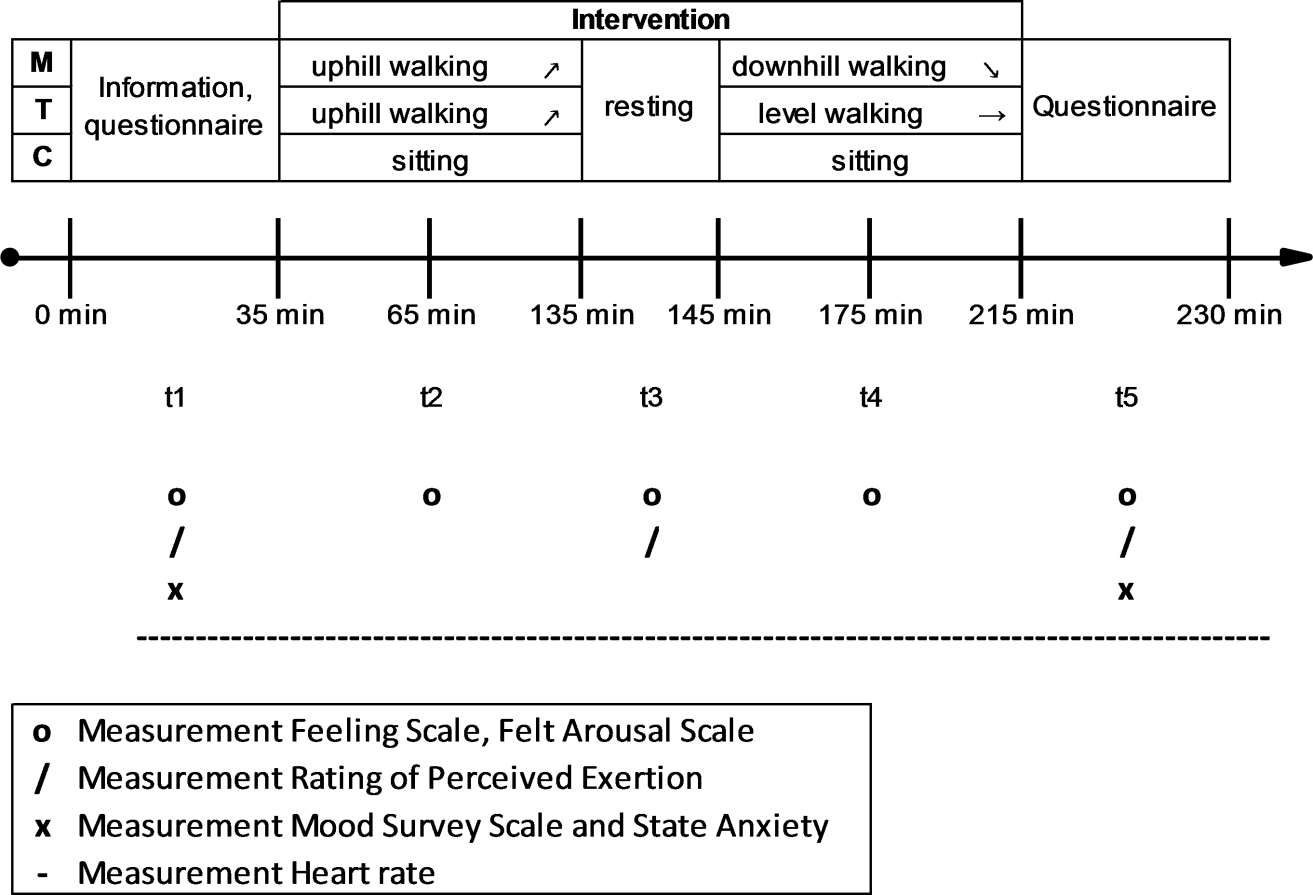
Sequence of events and timeline of conditions. M: outdoor mountain hiking, T: indoor treadmill walking, C: sedentary control condition.

In the first condition, the participants were informed about the procedure of the conditions and were asked to read and sign the consent form. To control for influences due to weather and daily constitution, we included a baseline measurement of affective states. After the baseline measurements (t1, compare measurements section) the first part of the intervention followed and lasted for approximately 100 minutes (see interventions section). After 10 minutes of rest (t3), the second part of the intervention lasted for approximately 70 minutes. At the end of the intervention (t5), the same measurements of t1 were repeated; the participants were instructed for the next condition and departed individually. The timeline remained the same in each condition, compare Fig 2.

Because affective responses can change within the PA condition [12], affective valence and activation were assessed five times: at t1, t2, t3, t4, and t5, respectively. Categorical affective responses were collected before (t1) and after the intervention (t5).

### Measurements

All measurements were performed in the group in a sedentary position with paper and pencil and supervised by MN. Four different sets of self-report questionnaires based on the Circumplex model [34] were used to assess affective responses, which can be operationalized by both a dimensional and categorical approach [35]. A dimensional approach covers basic dimensions of affect. Within the Circumplex model, two basic dimensions are used to operationalize affective responses, affective valence and perceived activation. Affective valence can be considered as the degree of pleasure (or displeasure) in a specific situation, while perceived activation is used for the feeling of arousal (high or low) [12].

Two single-item dimensional based scales to cover the two dimensions affective valence and arousal and two multi-item scales to cover nine selected distinctive states.

The Feeling Scale (FS) was used to operationalize affective valence (pleasure) [36]. The bipolar, single item scale consists of 11 answer possibilities ranging from “very good” (+5) to “very bad” (-5) with a neutral answer possibility. The FS was exclusively designed for measurements of affective responses during PA. Discriminant validity was reported for perceived exertion including other development information [36]. Convergent validity was assessed previously and is ranging from *r* = .41 to .88 [37].

Felt Arousal Scale (FAS) was used to assess perceived activation (high or low), which is usually described as orthogonal to affective valence [38]. Also designed as a bipolar, single-item scale, the FAS provides six response options ranging from “low arousal” (1) to “high arousal” (6). No neutral answer possibility is allowed. Van Landuyt & Ekkekakis [37] reported convergent validity with the arousal domain of the Self Assessment Manikin [39] or the Arousal Scale of the Affect Grid [40] (*r* = .45 to .70). In PA psychology, the combination of FS and FAS was used previously (e.g. [12]).

Because we were not only interested in dimensional changes, we also assessed distinct affective states in a categorical approach with a Mood Survey Scale (MSS) [41] and the German version of the State Trait Anxiety Inventory (STAI) [42], [43]. In the categorical approach, distinct affective states, e.g. anxiety, calmness, fatigue, or anger are distinguished. This approach was necessary to be able to gather differentiated information about possible changes in specific dimensions. The MSS is an adjective list of 40 items (“At this moment, I feel…”) with a five-point Likert response mode (“not at all” to “very”). Eight subscales (activation, elation, contemplation, calmness, fatigue, depression, anger, excitement) are calculated from five items each. Each subscale is ranging from five (lowest value) to 25 (highest value). Psychometric properties to convergent and divergent validity can be found in Abele-Brehm & Brehm [41]. Internal consistency ranges between Cronbach’s Alpha .70 and .88 [41]. The MSS can be compared with the Profile of Mood State [44], but has the advantage of providing four positive subscales instead of only one in the Profile of Mood State. The state subscale of the STAI was used for the measurement of state anxiety. The minimum value was 20 (low anxiety), the maximum value 80 (high anxiety). The German version of the STAI (state subscale) shows Cronbach’s Alpha for internal consistency between .90 and .94 [43]. Convergent validity and other psychometric values can be found in Laux & Glanzmann [43].

Finally, participants responded to the Rating of Perceived Exertion (RPE) to assess the perceived effort [45]. RPE was assessed on t1 with respect to the moment of assessment and on t3 and t5 retrospectively with respect to the corresponding intervention time. The RPE is ranging between six (“no exertion”) to 20 (“maximum exertion”). Validity information can be found at Borg [45] and Noble & Robertson [46].

All intervention time, heart rate (HR) was recorded using a wireless heart rate transmitter placed on a belt around the chest and a wristwatch (RS800CX, Polar Electro Oy, Finland). Additionally, heart rate was determined for five minutes in a sedentary position at t1 and t5. Average values of heart rate (t1, intervention time, t5) were used for the analysis.

Primary outcome parameters of the study were the affective states of FS and FAS, and the subscales of MSS and STAI. Secondary outcomes were the RPE and the heart rate.

### Interventions

#### Outdoor mountain hiking (M)

The outdoor mountain hiking condition was situated in a famous hiking area and started at the northern edge of Innsbruck with direct access to natural environment. The baseline measurements (t1) were conducted outdoors sitting on a bench (900 m). Directly after the measurements, the first part of the intervention started: an uphill hiking phase on single trails and forest roads in a sparse forest with view on the mountainous region around Innsbruck for six km in around 1.5 hours together with the test leader. Regarding the hiking intensity, the participants were instructed to choose a “brisk without overspending” pace (average speed: four km/h). The goal after uphill hiking was a mountain hut at 1500 m, where the participants responded to the RPE and rested for 10 minutes in a sedentary position (t3). In the second part of the intervention, the participants were hiking downhill on the same track for around 70 minutes back to the starting point to respond to the post-test (t5) with an average speed of 5.2 km/h. The participants were mainly hiking in subgroups of two to three people.

#### Indoor treadmill walking (T)

The indoor treadmill walking condition was situated in a fitness centre in Innsbruck. After recording the baseline measurements (t1), all participants were walking uphill on treadmills for the first part of the intervention. To ensure that all physical parameters were simultaneous to the outdoor mountain hiking condition, the distance, the difference in height, the average inclination of the track, and the time needed for the outdoor mountain hiking situation were measured in a pilot study. Consistent to that, the following settings were adjusted on the treadmill in the uphill situation: inclination: 10%, time: 1.5 hours, and speed: four km/h (resulting in 600 m difference in height). In accordance to possible differences in outdoor speed, the participants were allowed to change the treadmill’s speed in a small range (3.8 to 4.2 km/h) to adapt to the wording “brisk without overspending”. After responding to RPE and resting for 10 minutes (t3), the second part of the intervention contained 70 minutes of level walking on the same treadmills (5.2 km/h, six km). Unfortunately, downhill walking was not possible on the treadmills used. To simulate the group situation of the outdoor situation as good as possible, the treadmills were located side by side. After the second part of the intervention, participants responded to the post-test (t5).

#### Sedentary control condition (C)

The sedentary control situation was located in a quiet room at the university with access to computers. The participants were allowed to use the computers, to read, and to talk, but had to remain in a sedentary position. To control for possible differences in affective response due to the daytime, the sedentary control condition contained identical timing of the measurements as M and C. Sociodemographic data were collected for five to 10 minutes in this condition using a web-based questionnaire.

### Statistical analyses

All statistical analyses were performed using SPSS v. 23 (IBM, New York, United States). Possible systematic differences at t1 were tested by separate analyses of variances (AVONAs) with condition as within-factor (M—T—C).

Categorical affective responses (activation, elation, contemplation, calmness, fatigue, depression, anger, excitement) were analysed using three × two repeated measures ANOVAs with condition (M—T—C) and time (t1—t5) as within-factors. We used simple contrasts with outdoor mountain hiking as the reference condition to show significant interactions between M vs. C and M vs. T, respectively. Dependant variables were the subscale values of the MSS and STAI. We calculated Cohens d as an effect size [47]. Following the approach of Ensari & Greenlee [48], we calculated the mean change from before (t1) to after M (t5) and subtracted the mean change of C and T, respectively. The resulting difference was divided by the pooled standard deviation at t1. Cohens d can be classified in small (.3), middle (.5) and large (.8). Positive d-values mark a larger increase after M compared to T/C, negative values mark a larger decrease after M compared to T/C.

Concerning dimensional affective responses (affective valence and perceived activation), we used three × five repeated measures ANOVAs with condition (M—T—C) and time (t1—t2—t3—t4—t5) as within-factors. For the condition factor, we used simple contrasts as described above. For the time factor, we contrasted t1 as the reference time point. Dependant variables were the raw scores of FS and FAS. Since the level of physical activity might differently influence affective responses, all analyses were controlled for the covariate level of physical activity in an ANCOVA.

The level of significance was set at *p* < .05 (two-tailed). Bonferroni correction for multivariate analyses resulted in critical *p* values of *p* < .006 for the categorical approach and *p* < .025 for the dimensional approach.

Only M and T were analysed for differences in the secondary parameters, since it was expected that there will be differences between the physically active conditions and the sedentary condition. Possible differences between the conditions in perceived exertion were tested by using two × three repeated measures ANOVAs with condition (M—T) and time (t1—t3/intervention time—t5) as within-factors and with RPE and heart rate as the dependant variable.

Whenever the assumption of sphericity was not met in the ANOVA, Greenhouse-Geisser correction was applied. Unless otherwise stated, data was shown as mean (95% confidence interval).

## Results

### Preliminary analysis

The demographic data of the 42 participants (48% female) can be found in Table 1. There were no systematic differences between the conditions in any parameter at t1 (all *p* > .05). In all conditions, no harmful event for the participants happened.

**Table 1:** Demographic data of the study participants.

	Mean	95% CI				
Age (years)	32.0	(	28.4	-	35.6	)
Height (m)	1.74	(	1.71	-	1.77	)
Weight (kg)	69.0	(	65.7	-	72.3	)
Body mass index (kg/m^2^)	23.0	(	22.4	-	23.6	)
Physical activity (h/week)	8.0	(	6.5	-	9.5	)
Mountain tours (n/year)	27.2	(	19.3	-	35.1	)

95% CI: 95% Confidence Interval.

### Affective responses (dimensional)

**Fig 3:**
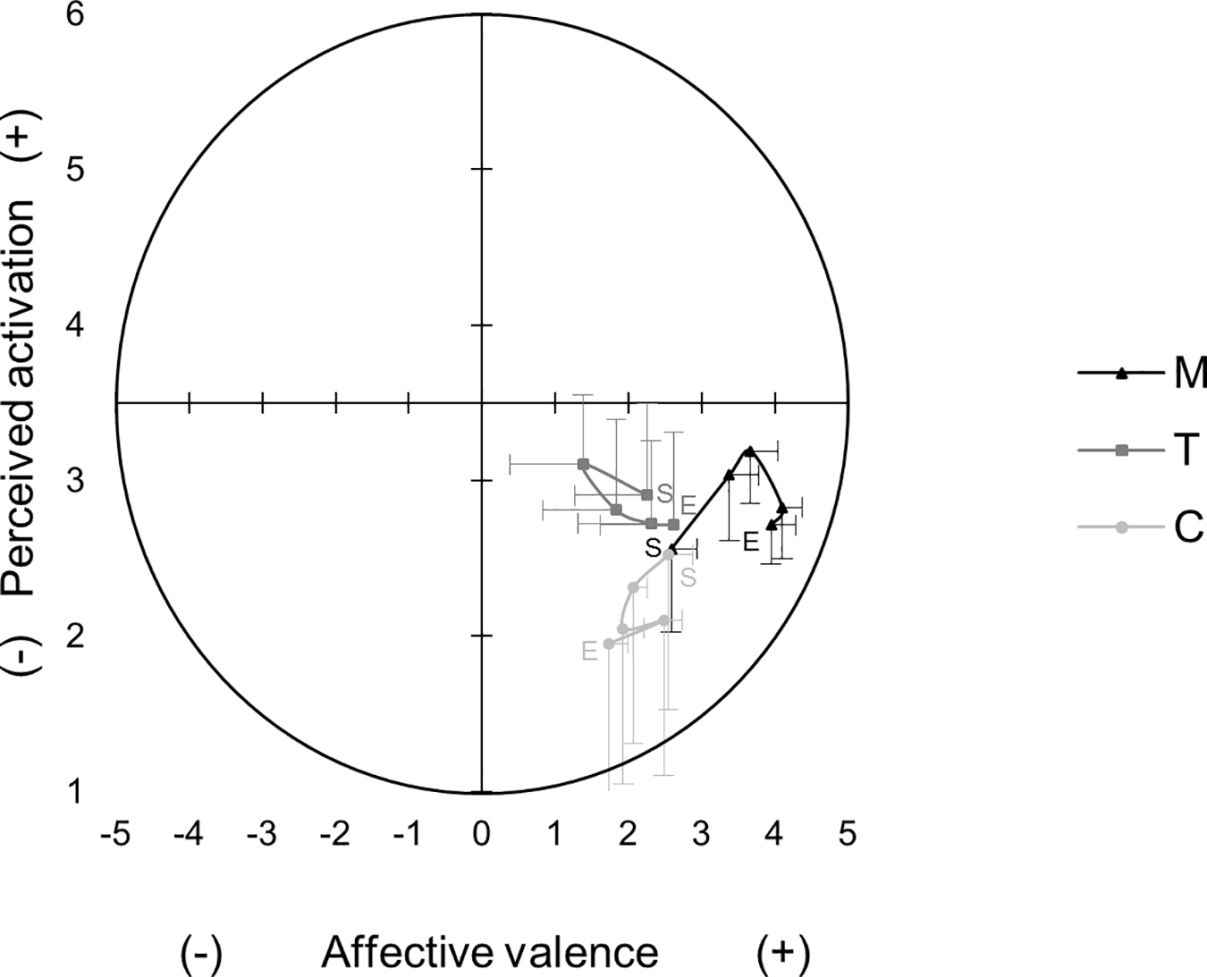
Mean affective valence and perceived activation over time in all conditions. M: outdoor mountain hiking, T: indoor treadmill walking, C: sedentary control condition, S: Start of the condition, E: End of the condition, error bars represent 95% CI.

Fig 3 shows the basic dimensions affective valence and perceived activation over all time points in all three conditions. Affective valence showed a significant interaction, *F*(5,147) = 6.30, *p* < .001, *d* = 1.21. In M vs. C, simple contrast calculation revealed significant interactions between t1 and all other time points (t2, t3, t4, t5), which indicated a different increase of affective valence between M and C from t1 to all other time points. In M vs. T, there were significant interactions between t1and t2, t4, t5. No significant interaction was observed between t1 and t3. Affective valence was rated with the highest values at t4 in condition M with the largest positive incline between t1 and t2. The largest decline in condition T was between t1 and t2.

There was no significant interaction in perceived activation, *p* > .025, *d* = .65, indicating a similar change in perceived activation between the conditions.

The covariate level of physical activity was not significant in the ANCOVA, all p > .05, indicating no significant influence of level of physical activity in our study.

### Affective responses (categorical)

**Table 2** shows the distinct states of the MSS subscale values and state anxiety (STAI) before and after each condition with the significance value for the interaction and the effect size for the contrasts. A significant interaction in activation, elation, calmness, fatigue, and anxiety was present, *F*(2,82) > 5.84, *p* < .005, |*d*| > .79. After M, positive affective states were increased and negative affective states were decreased. After C, the opposite effects of M were present in the according states.

**Table 2:** Affective responses (categorical) over time in each condition.

	M				T				C				p-value			d	
	t1		t5		t1		t5		t1		t5		condition	time	inter-action	M-C	M-T
	Mean	95% CI	Mean	95% CI	Mean	95% CI	Mean	95% CI	Mean	95% CI	Mean	95% CI					
Activation	16.3	(15.4–17.3)	16.9	(15.8–17.9)	16.4	(15.4–17.5)	15.1	(13.9–16.4)	15.7	(14.6–16.7)	13.4	(12.3–14.5)	< .001*	.007	.004*	.81	.54
Elation	18.0	(17.0–19.1)	19.5	(18.8–20.3)	17.3	(16.3–18.2)	17.6	(16.4–18.8)	18.0	(17.0–19.1)	15.8	(14.7–16.9)	< .001*	.727	< .001*	1.07	.33
Calmness	17.3	(16.3–18.3)	18.4	(17.5–19.2)	16.9	(15.9–17.9)	17.1	(15.9–18.2)	17.7	(16.8–18.5)	16.2	(15.2–17.2)	.063	.746	.004*	.84	.27
Fatigue	9.5	(8.3–10.6)	8.0	(7.1–8.8)	9.7	(8.4–11.0)	10.5	(9.0–12.0)	9.6	(8.5–10.7)	12.6	(11.2–13.9)	< .001*	.068	< .001*	-1.19	-.57
Depression	6.4	(5.6–7.2)	5.7	(5.3–6.1)	7.4	(6.6–8.1)	7.0	(6.1–7.9)	7.1	(6.2–8.0)	7.7	(6.8–8.5)	< .001*	.538	.094	-.43	-.12
Contemplation	9.9	(9.0–10.8)	9.9	(8.9–10.9)	10.2	(9.4–11.1)	9.5	(8.6–10.4)	10.8	(9.7–11.9)	10.4	(9.3–11.5)	.109	.125	.272	.15	.27
Anger	6.0	(5.4–6.6)	5.5	(5.2–5.8)	7.0	(6.3–7.6)	6.2	(5.5–7.0)	6.6	(6.0–7.2)	6.8	(6.1–7.6)	.008	.178	.105	-.36	.10
Excitement	8.8	(7.7–9.8)	6.8	(6.2–7.4)	9.2	(8.4–10.0)	8.1	(7.2–9.0)	8.6	(7.9–9.3)	7.9	(7.1–8.7)	.041	< .001*	.134	-.40	-.26
Anxiety (STAI)	37.5	(35.3–39.7)	33.6	(32.1–35.2)	38.6	(36.7–40.5)	36.6	(34.6–38.7)	37.0	(35.2–38.9)	38.4	(36.6–40.3)	.005*	.026	< .001*	-.79	-.28

M: outdoor mountain hiking, T: indoor treadmill walking, C: sedentary control condition; t1: pre intervention, t5: post intervention; STAI: State trait anxiety inventory; 95% CI: 95% Confidence Interval

* significance at p < .006; d: effect size for interaction contrasts, positive d-values mark a larger increase after M compared to T/C, negative values mark a larger decrease after M compared to T/C.

When M and T were contrasted, there was a significant interaction in activation and fatigue. Activation increased after M and decreased after T, while fatigue decreased after M and increased after T. Besides activation and fatigue, other subscales showed the same changes after T and M to a smaller extend.

The covariate level of physical activity was not significant in the ANCOVA, all p > .05, indicating no significant influence of level of physical activity in our study.

### Perceived exertion and heart rate

Mean RPE did not significantly differ between M and T across time points, *p* > .05, *d* = .17, indicating a similar change of perceived exertion between the physically active conditions.

There was a significant condition by time interaction in heart rate, *F*(2,80) = 7.6, *p* = .001, *d* = .59. Mean heart rate was differently changing between the conditions M and T. Repeated contrasts revealed significant differences between t1 vs. intervention time and intervention time vs. t5. During intervention time, mean HR was significantly higher in M compared to T, see **Table 3**. When expressed in percentage of the age predicted maximum heart rate after the formula of [49], mean HR in intervention time was 59 (57–63) % during condition M and 57 (54–59) % during condition T, respectively.

**Table 3:** Heart rate and self-reported physical exertion over time in each condition.

		M		T		C		p M-T
		Mean	95% CI	Mean	95% CI	Mean	95% CI	
HR (1/min)	t1	71.5	(67.7–75.4)	72.8	(69.3–76.3)	71.6	(67.8–75.5)	.001*
	Intervention time	110.9	(105.7–116.1)	105.0	(100.6–109.3)	68.7	(65.9–71.5)	
	t5	75.2	(69.4–81.0)	75.3	(69.8–80.8)	63.2	(60.4–66.0)	
RPE	t1	7.2	(6.5–7.9)	7.3	(6.7–7.8)	7.0	(6.5–7.4)	.573
	t3	11.0	(10.4–11.7)	11.5	(10.7–12.2)	7.5	(6.8–8.1)	
	t5	9.8	(9.3–10.4)	10.3	(9.7–11.0)	7.5	(6.7–8.2)	

M: outdoor mountain hiking, T: indoor treadmill walking, C: sedentary control condition; HR: heart rate, RPE: Rating of Physical Exertion; t1: pre intervention, t3: between intervention, t5: post intervention; 95% CI: 95% Confidence Interval

* significance at p < .05.

## Discussion

### Major findings

To the best of our knowledge, this was the first study to experimentally compare the acute effects on affective responses of an approximately three-hour PA intervention (mountain hiking) using a randomized crossover trial. The major findings of this study were that (1) a single bout of approximately three hours of outdoor PA (mountain hiking) can elicit significant positive changes in affective valence (pleasure) during and immediately after the intervention compared to a sedentary control situation. Immediately after PA, the positive affective states activation, elation, and calmness were increased, while the negative affective states fatigue and anxiety were decreased compared to the sedentary control situation. When comparing the conditions outdoor mountain hiking and indoor treadmill walking, our data showed that (2) green exercise resulted in a significantly larger increase of affective valence during and immediately after the intervention. Immediately after PA, outdoor mountain hiking showed significantly higher activation and significantly lower fatigue values compared to indoor treadmill walking. No significant interactions were observed in depression, anger, and excitement.

### Outdoor mountain hiking vs. sedentary control situation

Since this is the first study that seems to have observed the acute effects of a three-hour mountain hiking intervention, the possibility of comparing the current results with those of other studies is restricted. However, since mountain hiking is also a form of physical activity, the study can be compared to other forms of physical activity. The positive effects of a three-hour PA intervention in comparison to the sedentary control situation are similar to the reported effects of physical activity on affective responses with shorter duration previously reported. When compared to studies conducted in environments without altitude difference, not only findings from randomized controlled trials [29, 31, 50], but also findings from meta-analyses support the positive influence of physical activity on affective responses [28, 30, 51, 52]. When effect sizes were compared, the results of the present study (|*d|* = .79 to 1.21) showed even larger effect sizes than recently reported medium-sized effects by Barton & Pretty [28]: *d* = .56 or by Mackay & Neill [31]: *d* = .47. This discrepancy can partly be explained by different calculation methods. While Barton & Pretty [28] used the difference between pre- and post-intervention values, the calculation of the effects size was based on the interaction between intervention and control condition in the present study. Since the values for positive affect decreased and the values for negative affect increased in the control condition, this calculation method resulted in higher effect sizes in the present study. Furthermore, there were differences in the form of activity: Different activities (e.g. walking, cycling, gardening) were summarized in both the meta-analysis Barton & Pretty [28] and in the study of Mackay & Neill [31]. Finally, the measurement methods were different. Barton & Pretty [28] used the Profile of Mood State and Mackay & Neill [31] the State Trait Anxiety Inventory.

When the results are compared to studies using identical scale for affective valence (FS) and only walking as PA intervention, the effect sizes previously reported were lower (Ekkekakis & Backhouse [12]: *d* = .50 and .79) compared to the present values (*d* = 1.21). This was surprising, since Barton & Pretty [28] and Woo & Kim [22] stated a U-shaped curve of for the relationship between affective responses and PA duration showing the largest effect for five-to-10-minutes-bouts and the smallest for half-a-day bouts. Since PA intensity was comparable in both studies (age-predicted maximum heart rate: 67% vs. 64%), it might be speculated that a longer PA duration show larger effects on affective responses. However, the duration effect might be compensated by two other factors: While the mean age in the present study was 32 years, the mean age in the study of Ekkekakis & Backhouse [12] was 68 and 56 years, respectively. Barton & Pretty [28] reported a smaller effect size for older people, which could partly explain the difference between the two studies. Additionally, since it is known that walking in a pleasant surrounding can create larger effects on affective state [29], another reason for larger effects of mountain hiking compared to walking might come from the greenness of the environment and the view on the mountainous scenery, where the mountain hiking was located. This hypothesis is supported by the findings of Mackay & Neill [31], who stated an inverse relationship between the subjective greenness and the anxiety post PA.

We observed a discrepancy between perceived activation (dimensional approach), where no significant interaction was found, and activation (categorical approach), where we found a larger increase after mountain hiking compared to treadmill walking and sedentary control situation. An explanation might be found in differences in the meaning. Dimensional perceived activation is not related to the dimension of affective valence [39], i.e. perceived activation is measured with the absence of pleasure. In contrast to that, the items contributing to the categorical activation are positively connoted [41], i.e. with the presence of pleasure.

### Outdoor mountain hiking vs. indoor treadmill walking

We were postulating changes in affective responses due to (1) the PA and due to (2) being outdoors. Through the inclusion of the indoor treadmill walking situation we were able to show different effects of outdoor mountain hiking and indoor treadmill walking. After outdoor mountain hiking, the distinctive state activation was increased and fatigue was decreased with a medium sized effect compared to indoor treadmill walking. These results correspond to the findings of Pretty & Peacock [29] and of the meta-analysis of Thompson Coon & Boddy [30], who stated “greater feelings of revitalization and positive engagement” after physical activity outdoors. The authors concluded that a “natural environments may have beneficial effects on well-being” [30], which was supported by the results of the present study. As mentioned above, activation and fatigue of the MSS are tapping the energetic arousal dimension, which corresponds to greater feelings of revitalization and positive engagement. These results can be explained (1) by the mere exposure to nature, (2) by the effects of PA already mentioned, and (3) by the interaction of these variables. For the former, the psychophysiological stress recovery theory [53] states that the visual stimulus of nature itself elicits positive affective responses [54]. However, besides the differences in activation and fatigue, there were no significant differences between indoor treadmill walking and outdoor mountain hiking. Therefore, we conclude that the positive effects of PA on affective responses might be more important compared to the effects of the environment.

### Practical recommendations

From a public-health oriented perspective with increasing obesity rates [55], it is essential for health professionals to recommend efficient and pleasant forms of physical activity. According to the hedonic theory [56], people choose and maintain forms of physical activity they enjoy. Indeed, in a prospective study design, Williams & Dunsiger [14] reported that an increase of 1 point in the FS during physical activity would result in an increase of physical activity after six months of 40 minutes per week. Since outdoor mountain hiking increased mean affective valence from 2.6 to 4.0, mountain hiking can be recommended as a pleasant form of physical activity. When the results of Williams & Dunsiger [14] are applied to the present results, the amount of physical activity per week of the participants might be increased by around one hour in the future.

### Strengths and limitations

The following limitations have to be considered when interpreting the findings: Firstly, it was not feasible to apply downhill walking in the indoor situation, which resulted in a slightly different form of physical activity during the second part of the intervention. It remains therefore unknown if the differences between indoors and outdoors are only driven by the environment or also by the way of walking.

Secondly, affective responses might be influenced by at least two confounding variables: the level of social interaction and the intensity of PA. Although we provided a group setting in all conditions, we did not control for social interaction between the participants what might have affected the results. The heart rate during the intervention time in outdoor hiking was significantly higher than during indoor walking. Since mean inclination during uphill walking, duration, and speed were controlled, this discrepancy may have occurred by different changes in inclination in outdoor hiking, unequal surface and therefore a higher muscular activity in outdoor hiking or temperature differences between indoors and outdoors. According to the dual mode theory [21], this could have resulted in different affective responses. However, the rating of physical exertion was not significantly different.

Thirdly, we did not include a follow-up measurement. It is known that affective responses can change also up to 15 minutes after the walking intervention [12]. Future studies should consider a follow-up measurement.

Fourthly, we are aware about a possible selection bias in the present study. The participants were recruited on a voluntary basis without any compensation despite the amount of around 12 hours of workload. Therefore, an affinity to (mountain) sports cannot be ruled out in the participants of the present study, which could also explain the high amount of PA per week and the high amount of mountain tours per year. As we consider the provision of compensation also as a risk for a selection bias, we decided to select on a voluntary basis.

Fifthly, the length of the washout phase was not the same in all subjects due to conflicting schedules, which might have influenced the results. However, since there were no significant differences in baseline measurements before the conditions, we consider this as a minor limitation.

However, there are several strengths in the study to be mentioned: Firstly, the outdoor mountain hiking condition may be assumed to show high external validity because the applied hiking destination is a very popular and frequently visited one in the local area. Further, the applied outdoor hiking condition represents a typical duration, altitude difference and group size for recreational PA. Secondly, with the usage of a randomized crossover design, we were able to control for inter-individual variability by using the participants as their own controls. Thirdly, we were able to control for daily fluctuations by applying pre- and post- measurements.

## Conclusions

The findings of the present study indicate that an approximately three-hour PA intervention (mountain hiking) elicits higher positive and lower negative affective responses compared to a sedentary control situation. Therefore, also PA interventions with a longer duration (approximately three hours) can be recommended to improve affective responses. Additionally, the data suggest a synergetic effect of physical activity and being outdoors. These results provide support both for the psychophysiological stress recovery theory and for the affective benefits of green exercise. Thereby, similar previous findings of outdoor PA on state anxiety [31] and mood [29] were confirmed. Future research may include follow-up measurements and physiological assessments to verify the self-reported data.

## Supporting information

S1 Checklist CONSORT checklist. (PDF)

S1 Confirmation Institutional review board confirmation. (PDF)

S1 Dataset Data affective responses in mountain hiking. (SAV)

S1 Protocol Study protocol English. (PDF)

S2 Protocol Study protocol German. (PDF)
