# Supplementary material for: Affective responses in mountain hiking—A randomized crossover trial focusing on differences between indoor and outdoor activity
Source: PLoS One. 2017 May 16;12(5):e0177719. doi: 10.1371/journal.pone.0177719 (PMC5433751; doi:10.1371/journal.pone.0177719)
Supplement: S1 Confirmation — (PDF) [file pone.0177719.s002.pdf]

ISW

Fürstenweg 185
6020 Innsbruck

INSTITUT FÜR
SPORTWISSENSCHAFTEN
DER UNIVERSITÄT INNSBRUCK

universität innsbruck

Chairman
Univ.-Prof. Dr.
Elmar KORNEXL

Review Board for sports scientific research projects

Members:
Univ.-Prof. Dr. Elmar Kornexl
Univ.-Prof. DDr. Martin Burtscher
o.Univ.-Prof. Dr. Werner Nachbauer
Univ.-Ass. Dr. Barbara Hotter
Univ.-Prof. Dr. Martin Kopp
Univ.-Prof. Dr. Anna Buchheim

Innsbruck, *22.04.2015*
Zl. 025/2015

Documentation:

Project description:	*Effekte des Bergsports auf die individuelle Lebensqualität und Gesundheit*
Version/Date:	*22.04.2015*
Proband information:	yes
Version/Date:	*22.04.2015*
Cost report:	yes
Confirmation of insurance coverage (Probands/Testers):	yes
Necessary revision:	

Due to the presentation and documentation of the project of *Prof. Martin Kopp* there are no further objections to start this research project.

Chairman

E. Kornexl

Univ.-Prof. Dr. Elmar Kornexl

Institut für Sportwissenschaft d. Universität Innsbruck
