# Supplementary material for: Affective responses in mountain hiking—A randomized crossover trial focusing on differences between indoor and outdoor activity
Source: PLoS One. 2017 May 16;12(5):e0177719. doi: 10.1371/journal.pone.0177719 (PMC5433751; doi:10.1371/journal.pone.0177719)
Supplement: S1 Protocol — (PDF) [file pone.0177719.s004.pdf]

Study protocol English

Participant recruitment
Participant recruitment will be done on a voluntary basis by public announcements. Insurance will be taken out to cover possible accidents.

Inclusion criteria
- Age between 18 and 60 years
- Voluntary participation
- Ability to be physically active for a 3hours mountain hiking tour assessed by the Physical Activity Readiness Questionnaire (Shephard et al. 1991)

Exclusion criteria
- Pregnancy
- Breast-feeding
- Chronic or acute diseases (already existing or diagnosed during the study)

Sample size calculation
The required sample size was based on an a priori power analysis with the following assumptions. The effect size was set to $d = 1.3$, $\alpha = 0.05$, Power = 0.8 According to the power analysis, 25 subjects were necessary. A dropout rate of 20% was included and resulted in a minimum of 30 subjects.
To protect this calculation, a pilot study will be conducted and the effect size will be adapted accordingly.

Details of the interventions
(1) An outdoor mountain hiking condition with uphill and downhill walking phase around Innsbruck with duration of approximately 3 hours, approximately 700 altitude meters, and in a moderate walking intensity, RPE: 11-14.
(2) An indoor treadmill walking condition adapted to the outdoor mountain hiking condition regarding duration, inclination, intensity and rests.
(3) A sedentary control condition without physical activity with access to computers, RPE: 6. Duration is identical to the physical exercise conditions.

Outcome measures
Psychological: Mood survey scale (Befindlichkeitsskalen, Abele-Brehm et al. 1986; State Trait Anxiety Inventory, Spielberger et al. 1970) Feeling Scale (Hardy et al. 1989), Felt Arousal Scale (Svebak et al. 1985), Quality of life (World Health Organization 1998).
Physiological: Blood pressure, Saliva Sampling (Cortisol), Heart rate

Data collection
Psychological data will be collected in questionnaires at 3 (5 for Feeling Scale and Felt Arousal Scale) measurement points in each condition, before, at rest, and after the interventions.
Physiological data will be collected at 3 measurement points in each condition, before, at rest, and after the interventions.

Plans for data analysis
3 x 3 repeated measures ANOVAs (time by condition, both within-subject factors) or Friedman Test, as appropriate.
