# Supplementary material for: Affective responses in mountain hiking—A randomized crossover trial focusing on differences between indoor and outdoor activity
Source: PLoS One. 2017 May 16;12(5):e0177719. doi: 10.1371/journal.pone.0177719 (PMC5433751; doi:10.1371/journal.pone.0177719)
Supplement: S2 Protocol — (PDF) [file pone.0177719.s005.pdf]

Beirat für ethische Fragen in der wissenschaftlichen Forschung, Universität Innsbruck
(Geschäftsstelle: Büro der Vizerektorin für Forschung
Homepage: http://www.uibk.ac.at/rektorenteam/forschung/ethikbeirat.html)

An das
Büro der Vizerektorin für Forschung der Universität Innsbruck
zu Handen des betreffenden Review Boards der Fakultät

Review Board „Sportwissenschaften“<br>Institut für Sportwissenschaften<br>z.Hd. Fr. Andrea Hofer<br>@: Andrea.Hofer@uibk.ac.at	Review Board „Psychologie“<br>Institut für Psychologie<br>z.Hd. Fr. Univ.-Prof. Dr. Anna Buchheim<br>@: Anna.Buchheim@uibk.ac.at	Review Board „Sozialwissenschaften“<br>Fakultät für Betriebswirtschaft und Fakultät für Volkswirtschaft und Statistik<br>z.Hd. Hr. Univ.-Prof. Mag. Dr. Michael Kirchler<br>@: Michael.Kirchler@uibk.ac.at

Eingangsvermerk: …………………

Erledigungsvermerk: ………………

Ansuchen um Prüfung der Unbedenklichkeit eines Forschungsprojektes

Name des/der Projektleiters/Projektleiterin: Univ.-Prof. Mag. Dr. Martin Kopp

Projekttitel: Effekte des Bergsports auf die individuelle Lebensqualität und Gesundheit

Institut: Institut für Sportwissenschaft

Datum: 03.03.2015

Projektbeschreibung – Effekte des Bergsports auf die individuelle Lebensqualität und Gesundheit

Wissenschaftlicher Hintergrund und Neuwert:

Auswirkungen von physischer Aktivität auf die körperliche und zunehmend auch psychische Gesundheit werden in der Literatur eingehend untersucht (Paffenbarger et al. 1978, Reddy 2004, Penedo et al. 2005, Lees et al. 2013, Deslandes 2014). Bisher vorliegende Interventionsstudien haben meist die Wirkung von aerobem Ausdauersport ohne die Überwindung von Höhenunterschieden (z.B. Radfahren, Wandern in der Ebene, Spielsportarten) untersucht (Yeung 1996). Sowohl auf die physische als auch auf die psychische Gesundheit konnten positive Effekte gezeigt werden. So reduziert körperliche Aktivität Angst (Petruzzello et al. 1991) und Depression (North et al. 1990) und zeigt positive Effekte auf die Befindlichkeit (Yeung 1996).

Neben den oben genannten Sportarten spielt bei Menschen aus Alpenländern der Bergsport eine zentrale Rolle (Bourdeau et al. 2002). Der Umstand, dass sich die Berge direkt vor der Haustüre befinden, lädt viele Menschen dazu ein, Sportarten wie beispielsweise Bergwandern, Hochtouren gehen, Skitourengehen, Mountainbiking und weitere auszuüben. Die Anziehungskraft der Berge beschränkt sich jedoch nicht nur auf Menschen aus Ländern mit Bergregionen. Neben dem Meer sind die Berge eine beliebte Urlaubsdestination. Allein in den Alpen werden jährlich 40 Millionen Bergtouristen gezählt (Burtscher 1999).

Die physische Aktivität beim Bergsport unterscheidet sich jedoch von der physischen Aktivität ohne die Überwindung von Höhenunterschieden hinsichtlich der äußeren (z.B. Temperaturveränderung) und inneren Faktoren (z.B. Art der Muskelarbeit). Es stellt sich also die Frage, ob der Bergsport vergleichbare Effekte auf individuelle Lebensqualität und Gesundheit zeigt. Bisher wurde zu dieser Thematik nur eine Pilotstudie an Suizidpatienten durchgeführt (Sturm et al. 2012). Zu gesunden Probanden sind noch keine Daten vorliegend.

Zielsetzung:

Ziel der vorliegenden Arbeit ist, es herauszufinden, welche unmittelbaren und chronischen Auswirkungen der Bergsport auf die individuelle Lebensqualität, die psychische Gesundheit und die körperliche Beschwerdewahrnehmung hat.

Methodik:

Das Projekt besteht aus den 3 Teilprojekten: A, B und C. Potentielle akute Effekte werden in Teilprojekt A und B untersucht, etwaige chronische Effekte werden in Teilprojekt C behandelt.

Teilprojekt A besteht für alle Teilnehmenden neben einer Anamnese aus 3 Bedingungen, die randomisiert zugeteilt werden und in einem Gesamtzeitraum von 3 Wochen stattfinden: Bergwanderung, Laufbandeinheit (je moderate Intensität), Kontrollsituation. Vor, während und nach den Bedingungen werden Fragebogenerhebungen (Lebensqualität, Befindlichkeit, Stressempfinden, Depression, Angst, körperliches Beschwerdeempfinden) sowie physiologische Messungen (Speichelproben, Kapillarblut, Herzfrequenz, Blutdruck) durchgeführt.
In Teilprojekt B werden andere Probanden als In Teilprojekt A untersucht. Es besteht für alle Teilnehmenden aus 2 Bedingungen, die randomisiert zugeteilt werden: Bergwanderung in Räumen mit großem anthropogenem Einfluss, Bergwanderung in Räumen mit geringem anthropogenen Einfluss (moderate Intensität). Vor, während und nach den Bedingungen werden die gleichen Fragebogenerhebungen wie in Teilprojekt A durchgeführt. Es werden keine physiologischen Parameter erhoben.
Teilprojekt C ist eine Online-Fragebogenerhebung. Es werden Bergaktive mit der österreichischen Bevölkerung verglichen und Unterschieds- und Korrelationsanalysen durchgeführt. Dazu wird das Bewegungsverhalten, die Lebensqualität, die Befindlichkeit und Arbeitsfähigkeit erfasst.
Die einzelnen Fragebögen sind der Case Report Form zu entnehmen.

<u>Abwägung des Nutzen-Risiko-Verhältnisses</u>
Bergwanderungen in mittleren Höhenlagen sind im Vergleich zu anderen Sportarten mit einem geringen Risiko assoziiert. So gibt das Kuratorium für Verkehrssicherheit in Österreich an, dass sich 4% der Unfälle beim Wandern und Bergsteigen ereignen (Kuratorium für Verkehrssicherheit 2013). Dagegen steht beispielsweise der alpine Skilauf, bei dem sich über 20% gesamten Unfälle in Österreich ereignen. Die moderate Intensität und die geringe Dauer der Intervention in der geplanten Studie reduzieren das Risiko zusätzlich.
Gleichzeitig gibt es eine große Anzahl von Personen, die Bergsport ausüben (Burtscher 1999). Wissenschaftlich sind aber die gesundheitlichen Auswirkungen des Bergsports und insbesondere des Bergwanderns kaum systematisch untersucht worden. Daher ist diese Untersuchung von großer gesundheitssportlicher und präventiver Bedeutung.
Teilprojekt A und B: Es sind keine besonderen Risiken abseits einer normalen Bergwanderung zu erwarten.
Teilprojekt C: Es sind keine besonderen Risiken abseits einer Fragebogenerhebung zu erwarten.

<u>Globalbudget und Drittmittel</u>
Drittmittel des Österreichischen Alpenvereins: EUR 92.000, davon EUR 72.000 an die Leopold-Franzens-Universität Innsbruck und EUR 20.000 an die Paracelsus Medizinische Privatuniversität Salzburg. Kosten Dissertantenstelle: EUR 72.000, Kosten Versicherung: etwa EUR 500, Kosten Auswertung Paracelsus Medizinische Privatuniversität Salzburg: EUR 20.000. Es handelt sich um Forschungsförderung, keine Auftragsforschung.

<u>Fallzahlschätzung</u>
Teilprojekt A: n=30, Teilprojekt B: n=60, Teilprojekt C: n=2000.
Für die Kalkulation der Stichprobengröße wurden Poweranalysen unter folgenden Annahmen durchgeführt:
A: Nach akuter Bewegungsintervention berichten Hamer et al. (2005) in ihrer Metaanalyse eine Gesamteffektstärke von d=0,40 für die Reduktion physiologischer Stressparameter. Dieser Effekt erhöht sich, wenn die Bewegung länger andauert (1h: d=0,99). Bei einer Bewegungsintervention von etwa 3 Stunden wird daher mit einem großen Effekt von d=1,3 gerechnet. Um einen Effekt von d=1,3 bei $\alpha=0{,}05$ und $1-\beta=0{,}8$ nachzuweisen, sind für drei Messzeitpunkte mindestens 25 Probanden notwendig. Wenn eine Dropout-Rate von 20% mit einbezogen wird, ergibt sich ein Mindestumfang von 30 Probanden. Um diese Kalkulation abzusichern wird eine Voruntersuchung durchgeführt.
B: Thompson Coon et al. (2011) beschreiben in ihrem Review über die Unterschiede bei körperlicher Aktivität indoor bzw. outdoor einen mindestens moderaten Effekt auf die Befindlichkeit zu Gunsten der Aktivität outdoor (d=0,42). Unterschiede zwischen angenehm und unangenehm wahrgenommener Umgebung outdoor untersuchten Pretty et al. (2005). Die Effektstärken reichen je nach Subskala der Befindlichkeit von d=0,41 (Angst) bis d=1,75 (Depression). Um einen Effekt von d=0,41 bei $\alpha=0{,}05$ (zweiseitig) und $1-\beta=0{,}8$ nachzuweisen, sind mindestens 49 Probanden notwendig. Wenn eine Dropout-Rate von 20% mit einbezogen wird, ergibt sich ein Mindestumfang von 60 Probanden.
C: Da es bei diesem Projekt um einen möglichst umfassenden Einblick in das Bewegungs- und Bergsportverhalten sowie die Lebensqualität von bergsportaktiven Personen und zudem diverse Subgruppenanalysen geplant sind, wurde keine Poweranalyse durchgeführt.

<u>Versicherung der ProbandInnen</u>
Für Teilprojekt A wird eine Personenunfallversicherung abgeschlossen. Teilprojekt B wird im Rahmen von einer gewöhnlichen Sektionsveranstaltung abgehalten. Etwaige Personenschäden sind daher mit dem Versicherungsschutz des Österreichischen Alpenvereins gedeckt, was mit dem Ansprechpartner beim Alpenverein bereits abgesprochen ist.
Da Teilprojekt C eine Online Fragebogenerhebung ist, ist keine Versicherung vonnöten.

Nachweis der Qualifikationen des Studienleiters

Als Nachweis sind Publikationen des Studienleiters aufgelistet.

Ledochowski L, Kopp-Wilfling P, Schwerdtfeger A, Höfer S; **Kopp M.** Bewegung und psychisches Wohlbefinden: Affektive Reaktionen auf körperliche Aktivität. Psychologie in Österreich 2/3 2014; 184-192.

Ledochowski L, Taylor AH, Haasova M, Faulkner GE, Ussher MH, **Kopp M.** Unmittelbare Auswirkungen einzelner Bewegungseinheiten auf das Bedürfnis zu rauchen– ein systematischer Literaturüberblick. Zeitschrift für Gesundheitspsychologie, 2013 21 (3), 122 – 137.

Kopp M, Steinlechner M, Ruedl G, Ledochowski L, Rumpold G, Taylor AH. Acute effects of brisk walking on affect and psychological well-being in individuals with type 2 diabetes. Diabetes Research and Clinical Practice 2012 Jan;95(1):25-9.

Stark R, Schöny W, **Kopp M.** Auswirkungen einer moderaten Bewegungseinheit auf die psychische Befindlichkeit bei PatientInnen mit affektiven Störungen in stationär psychiatrischer Behandlung. Neuropsychiatr. 2012 Oct 31. [Epub ahead of print] German.

Pfaffenberger N, Doering S, Puffinger P, Höfer S, Alber H, Ruttmann E, Günther V, **Kopp M**. Health-related quality of life, anxiety and depression before and after coronary artery bypass grafting. Wien Med Wochenschr. 2010 Jan; 160(1-2):44-53.

Kopp M. Körperliche Aktivität bei Menschen mit schweren psychischen Erkrankungen: Stand der Forschung und praktische Empfehlungen. Neuropsychiatr. 2009;23(3):151-6.

Unterschrift:

…………………………………………………………..
(Projektleiterin/Projektleiter)

Anlagen:

A) Proband/inn/eninformation und Einwilligungserklärung für ProbandInnen A

Warum wird die Studie durchgeführt?

Die Studie wird durchgeführt um die akuten Effekte von Bergsport auf die psychische Gesundheit sowie das Beschwerdeempfinden zu erfassen.

Wer darf an der Studie teilnehmen?

Es werden Probanden aus dem Raum Innsbruck rekrutiert. Ausschlusskriterien sind akute Schwangerschaft, Stillperiode, bestehende oder im Verlauf der Untersuchungen diagnostizierte chronische oder akute Erkrankungen. Einschlusskriterien sind Alter zwischen 18 und 60 Jahren und Fähigkeit zu einer 3stündigen Bergwanderung.

Wer führt die Studie durch?

Die Studie ist eine Kooperation des Österreichischen Alpenvereins, der Paracelsus Medizinische Privatuniversität Salzburg und der Leopold-Franzens-Universität Innsbruck.
Studienleitung: Univ.-Prof. Dr. Martin Kopp.
Studienkoordination: Martin Niedermeier.

Versuchsablauf

Der Versuchsablauf besteht für alle Teilnehmenden neben einer Anamnese aus 3 Bedingungen, die randomisiert zugeteilt werden und in einem Gesamtzeitraum von 3 Wochen stattfinden: Bergwanderung, Laufbandeinheit (je moderate Intensität), Kontrollsituation. Vor, während und nach den Bedingungen werden Fragebogenerhebungen (Befindlichkeit, Stress, Depression, Angst, Beschwerdeempfinden) sowie physiologische Messungen (Speichelproben, Herzfrequenz, Blutdruck) durchgeführt. Der Zeitbedarf beträgt ca. 4 Stunden pro Bedingung. Die Gesamtlaufzeit der Studie ist bis Ende 2016 konzipiert. Es sind keine besonderen Risiken abseits einer normalen Bergwanderung zu erwarten

Versicherung

Es wird eine Personenunfallversicherung abgeschlossen.

Datenschutz

Personenbezogene Daten dürfen im Rahmen der Studie nur mit ausdrücklicher Zustimmung des Betroffenen verwendet werden. Im Regelfall haben nur der Studienleiter und dessen Mitarbeiter sowie die Mitarbeiter in- und ausländischer Gesundheitsbehörden, an welche die Daten übermittelt wurden, Zugang zu diesen personenbezogenen Daten. Alle Personen, die aufgrund ihrer beruflichen Tätigkeit Zugang zu diesen Daten haben, sind, unbeschadet anderer gesetzlicher Verschwiegenheitspflichten, gemäß § 15 DSG 2000 an das Datengeheimnis gebunden. Die Übermittlung der Daten im In- und Ausland erfolgt im Übrigen ausschließlich zu statistischen Zwecken und in pseudonymisierter Form. Allfällige Veröffentlichungen der Daten dieser Studie erfolgen in anonymisierter Form. Auch wenn Sie ohne Zwang und in Kenntnis der Sachlage für den konkreten Fall der Verwendung Ihrer personenbezogenen Daten im Rahmen dieser Untersuchung zugestimmt haben, steht Ihnen die Möglichkeit offen, diese Zustimmung jederzeit ohne Angabe von Gründen zu widerrufen. Der Widerruf wird im Regelfall mit Ihrem Ausscheiden aus der Studie verbunden sein.

Freiwilligkeit

Ihre Teilnahme an dieser Studie erfolgt freiwillig. Sie können jederzeit ohne Angabe von Gründen aus der Studie ausscheiden. Die Ablehnung der Teilnahme oder ein vorzeitiges Ausscheiden aus dieser Studie hat keine nachteiligen Folgen für Sie. Ebenso kann von Seiten des Studienleiters Ihre Teilnahme an der Studie vorzeitig beendet werden. Wenn die Aufklärung und Information

vollkommen zufriedenstellend sind, bitten wir um Unterzeichnung der nachfolgenden Einverständniserklärung.

Einverständniserklärung

Ich habe die Patienteninformation und Einverständniserklärung gelesen und verstanden. Alle meine Fragen wurden beantwortet und ich habe zurzeit keine weiteren Fragen mehr. Sollten sich während der Studie Fragen ergeben, kann ich mich jederzeit an Herrn Dr. Martin Kopp (Institut für Sportwissenschaft der Universität Innsbruck, Fürstenweg 185, A-6020 Innsbruck, Tel. +43 (0)512 507 45866, Martin.Kopp@uibk.ac.at) oder die Mitarbeiter der Studie wenden.

Nach der datenschutzrechtlichen Aufklärung erteile ich meine ausdrückliche Zustimmung, dass meine personenbezogenen Daten im Rahmen der Studie „Effekte des Bergsports auf die individuelle Lebensqualität und Gesundheit" zu dem angeführten Studienzweck verarbeitet werden.

Ich bestätige mit meiner Unterschrift, dass ich freiwillig und aus eigenem Interesse an dieser Studie teilnehme.

Innsbruck, ____________________
Ort, Datum

Innsbruck, __________________
Ort, Datum

____
Name des Studienteilnehmers

____
Studienkoordination

____
Unterschrift des Studienteilnehmers

____
Unterschrift der Studienkoordination

B) Proband/inn/eninformation und Einwilligungserklärung für ProbandInnen B

Warum wird die Studie durchgeführt?

Die Studie wird durchgeführt um die akuten Effekte von Bergsport auf die psychische Gesundheit sowie das Beschwerdeempfinden zu erfassen.

Wer darf an der Studie teilnehmen?

Es werden Probanden in Sektionsveranstaltungen des Österreichischen Alpenvereins rekrutiert. Ausschlusskriterien sind akute Schwangerschaft, Stillperiode, bestehende oder im Verlauf der Untersuchungen diagnostizierte chronische oder akute Erkrankungen. Einschlusskriterien sind Alter zwischen 18 und 60 Jahren und Teilnahme am Kursangebot des Österreichischen Alpenvereins.

Wer führt die Studie durch?

Die Studie ist eine Kooperation des Österreichischen Alpenvereins, der Paracelsus Medizinische Privatuniversität Salzburg und der Leopold-Franzens-Universität Innsbruck.
Studienleitung: Univ.-Prof. Dr. Martin Kopp.
Studienkoordination: Martin Niedermeier.

Versuchsablauf

Der Versuchsablauf besteht für alle Teilnehmenden aus einer Anamnese (Teil 1 und 2) sowie 2 bergsportlichen Unternehmungen. Vor, während und nach den Unternehmungen werden Fragebogenerhebungen zur Befindlichkeit und dem körperlichen Beschwerdeempfinden (Teil 3, 4 und 5) durchgeführt, wobei die gleichen Fragebögen mehrmals zum Einsatz kommen. Es sind keine besonderen Risiken abseits einer normalen Bergwanderung zu erwarten

Datenschutz

Personenbezogene Daten dürfen im Rahmen der Studie nur mit ausdrücklicher Zustimmung des Betroffenen verwendet werden. Im Regelfall haben nur der Studienleiter und dessen Mitarbeiter sowie die Mitarbeiter in- und ausländischer Gesundheitsbehörden, an welche die Daten übermittelt wurden, Zugang zu diesen personenbezogenen Daten. Alle Personen, die aufgrund ihrer beruflichen Tätigkeit Zugang zu diesen Daten haben, sind, unbeschadet anderer gesetzlicher Verschwiegenheitspflichten, gemäß § 15 DSG 2000 an das Datengeheimnis gebunden. Die Übermittlung der Daten im In- und Ausland erfolgt im Übrigen ausschließlich zu statistischen Zwecken und in pseudonymisierter Form. Allfällige Veröffentlichungen der Daten dieser Studie erfolgen in anonymisierter Form. Auch wenn Sie ohne Zwang und in Kenntnis der Sachlage für den konkreten Fall der Verwendung Ihrer personenbezogenen Daten im Rahmen dieser Untersuchung zugestimmt haben, steht Ihnen die Möglichkeit offen, diese Zustimmung jederzeit ohne Angabe von Gründen zu widerrufen. Der Widerruf wird im Regelfall mit Ihrem Ausscheiden aus der Studie verbunden sein.

Freiwilligkeit

Ihre Teilnahme an dieser Studie erfolgt freiwillig. Sie können jederzeit ohne Angabe von Gründen aus der Studie ausscheiden. Die Ablehnung der Teilnahme oder ein vorzeitiges Ausscheiden aus dieser Studie hat keine nachteiligen Folgen für Sie. Ebenso kann von Seiten des Studienleiters Ihre Teilnahme an der Studie vorzeitig beendet werden. Wenn die Aufklärung und Information vollkommen zufriedenstellend sind, bitten wir um Unterzeichnung der nachfolgenden Einverständniserklärung.

Einverständniserklärung

Ich habe die Patienteninformation und Einverständniserklärung gelesen und verstanden. Alle meine Fragen wurden beantwortet und ich habe zurzeit keine weiteren Fragen mehr. Sollten sich während der Studie Fragen ergeben, kann ich mich jederzeit an Herrn Dr. Martin Kopp (Institut für

Sportwissenschaft der Universität Innsbruck, Fürstenweg 185, A-6020 Innsbruck, Tel. +43 (0)512 507 45866, Martin.Kopp@uibk.ac.at) oder die Mitarbeiter der Studie wenden.
Nach der datenschutzrechtlichen Aufklärung erteile ich meine ausdrückliche Zustimmung, dass meine personenbezogenen Daten im Rahmen der Studie „Effekte des Bergsports auf die individuelle Lebensqualität und Gesundheit“ zu dem angeführten Studienzweck verarbeitet werden.

Ich bestätige mit meiner Unterschrift, dass ich freiwillig und aus eigenem Interesse an dieser Studie teilnehme.

______________________________	______________________________
Ort, Datum	Ort, Datum
______________________________	______________________________
Name des Studienteilnehmers	Studienkoordination
______________________________	______________________________
Unterschrift des Studienteilnehmers	Unterschrift der Studienkoordination

C) Case Report Form A (Dokumentationsbogen für die zu erhebenden Daten)

Teil 1: Soziodemographische Daten

Die folgenden Fragen beziehen sich auf Ihre Person. Sollten Sie sich bei einer Frage nicht sicher sein, lassen Sie bitte die Frage aus und gehen Sie über zur nächsten Frage.

1.1	Bitte geben Sie ihr Geschlecht an.<br>☐ weiblich<br>☐ männlich
1.2	Bitte geben Sie Ihr Alter an.<br>_______ Jahre
1.3	Bitte geben Sie Ihre Größe an.<br>_______ Zentimeter
1.4	Bitte geben Sie Ihr Gewicht an.<br>_______ Kilogramm
1.5	Bitte geben Sie Ihre Nationalität an.<br>______________________
1.6	Bitte geben Sie Ihre Postleitzahl an.<br>______________________
1.7	Wie viele Stunden pro Woche betreiben Sie durchschnittlich Sport?<br>_______ Stunden pro Woche
1.8	Welche Sportarten betreiben Sie regelmäßig (durchschnittlich mind. 1x pro Woche)?<br>1.__________________<br>2.__________________<br>3.__________________<br>(Wenn Sie keine Sportart betreiben, lassen Sie die Frage aus.)
1.9	Wie viele Bergtouren (Wandern, Skitouren, Rodeln, Hochtouren etc.) unternehmen Sie durchschnittlich pro Jahr?<br>_______ Touren pro Jahr
1.10	Sind Sie Mitglied bei einem alpinen Verein?<br>☐ nein<br>☐ ja<br>wenn ja, bei welchem:<br>☐ Österreichischer Alpenverein<br>☐ Deutscher Alpenverein<br>☐ Schweizer Alpenclub<br>☐ Alpenverein Südtirol<br>☐ Sonstige
1.11	Was ist Ihr gegenwärtiger Familienstand?<br>☐ verheiratet/in Partnerschaft lebend<br>☐ alleinstehend<br>☐ geschieden<br>☐ verwitwet<br>☐ Sonstiges
1.12	Was ist Ihre höchste abgeschlossene Schulbildung?<br>☐ Kein Abschluss<br>☐ Pflichtschule (Hauptschule, Polytechnische Lehranstalt)<br>☐ Abgeschlossene Lehre<br>☐ Matura / Abitur (AHS/BHS)<br>☐ Universität, Fachhochschule

1.13	Welche berufliche Stellung haben Sie derzeit? (Hauptberuf)<br>☐ SchülerIn<br>☐ StudentIn<br>☐ Angestellte(r)<br>☐ Beamter/in<br>☐ Hausmann/Hausfrau<br>☐ Nicht erwerbstätig: Arbeitssuchend<br>☐ Nicht erwerbstätig: In Rente/Pension<br>☐ Selbstständig/Freier Dienstnehmer<br>☐ Sonstiges
1.14	Um die Fragebögen der einzelnen Messzeitpunkte einander zuordnen zu können, wird ein Kürzel benötigt. Bitte geben Sie den Anfangsbuchstaben des Vornamens Ihrer Mutter das Geburtsjahr Ihrer Mutter sowie den Anfangs- und Endbuchstaben Ihres Vornamens an. (Beispiel: Mutter: Johanna, geboren 1960, Vorname Max, = j1960mx)<br>____________________

Teil 2: Einschätzung Lebensqualität

Bitte lesen Sie jede Frage, überlegen Sie, wie Sie sich in den vergangenen **zwei Wochen** gefühlt haben, und kreuzen Sie das Kästchen auf der Skala an, das für Sie am ehestens zutrifft. Wenn Sie sich bei der Beantwortung einer Frage nicht sicher sind, wählen Sie bitte die Antwortkategorie, die Ihrer Meinung nach am ehesten zutrifft. Oft ist dies die Kategorie, die Ihnen **als erstes** in den Sinn kommt.

		sehr schlecht	schlecht	mittel-mäßig	gut	sehr gut
2.1	Wie würden Sie Ihre Lebensqualität beurteilen?	☐	☐	☐	☐	☐

		sehr un-zufrieden	un-zufrieden	weder zufrieden noch un-zufrieden	zufrieden	sehr zufrieden
2.2	Wie zufrieden sind Sie mit Ihrer Gesundheit?	☐	☐	☐	☐	☐

In den folgenden Fragen geht es darum, wie stark Sie während der vergangenen zwei Wochen bestimmte Dinge erlebt haben.

		überhaupt nicht	ein wenig	mittel-mäßig	ziemlich	äußerst
2.3	Wie stark werden Sie durch Schmerzen daran gehindert, notwendige Dinge zu tun?	☐	☐	☐	☐	☐
2.4	Wie sehr sind Sie auf medizinische Behandlung angewiesen, um das tägliche Leben zu meistern?	☐	☐	☐	☐	☐
2.5	Wie gut können Sie Ihr Leben genießen?	☐	☐	☐	☐	☐
2.6	Betrachten Sie Ihr Leben als sinnvoll?	☐	☐	☐	☐	☐
2.7	Wie gut können Sie sich konzentrieren?	☐	☐	☐	☐	☐
2.8	Wie sicher fühlen Sie sich in Ihrem täglichen Leben?	☐	☐	☐	☐	☐
2.9	Wie gesund sind die Umweltbedingungen in Ihrem Wohngebiet?	☐	☐	☐	☐	☐

In den folgenden Fragen geht es darum, in welchem Umfang Sie während der vergangenen zwei Wochen bestimmte Dinge erlebt haben oder in der Lage waren, bestimmte Dinge zu tun.

		überhaupt nicht	ein wenig	mittel-mäßig	ziemlich	äußerst
2.10	Haben Sie genug Energie für das tägliche Leben?	☐	☐	☐	☐	☐
2.11	Können Sie Ihr Aussehen akzeptieren?	☐	☐	☐	☐	☐
2.12	Haben Sie genug Geld, um Ihre Bedürfnisse erfüllen zu können?	☐	☐	☐	☐	☐
2.13	Haben Sie Zugang zu den Informationen, die Sie für das tägliche Leben brauchen?	☐	☐	☐	☐	☐
2.14	Haben Sie ausreichend Möglichkeiten zu Freizeitaktivitäten?	☐	☐	☐	☐	☐

		sehr schlecht	schlecht	mittel-mäßig	gut	sehr gut
2.15	Wie gut können Sie sich fortbewegen?	☐	☐	☐	☐	☐

In den folgenden Fragen geht es darum, wie zufrieden, glücklich oder gut Sie sich während der vergangenen zwei Wochen hinsichtlich verschiedener Aspekte Ihres Lebens gefühlt haben.

		sehr un-zufrieden	un-zufrieden	weder zufrieden noch un-zufrieden	zufrieden	sehr zufrieden
2.16	Wie zufrieden sind Sie mit Ihrem Schlaf?	☐	☐	☐	☐	☐
2.17	Wie zufrieden sind Sie mit Ihrer Fähigkeit, alltägliche Dinge erledigen zu können?	☐	☐	☐	☐	☐
2.18	Wie zufrieden sind Sie mit Ihrer Arbeitsfähigkeit?	☐	☐	☐	☐	☐
2.19	Wie zufrieden sind Sie mit sich selbst?	☐	☐	☐	☐	☐
2.20	Wie zufrieden sind Sie mit Ihren persönlichen Beziehungen?	☐	☐	☐	☐	☐
2.21	Wie zufrieden sind Sie mit Ihrem Sexualleben?	☐	☐	☐	☐	☐
2.22	Wie zufrieden sind Sie mit der Unterstützung durch Ihre Freunde?	☐	☐	☐	☐	☐
2.23	Wie zufrieden sind Sie mit Ihren Wohnbedingungen?	☐	☐	☐	☐	☐
2.24	Wie zufrieden sind Sie mit Ihren Möglichkeiten, Gesundheitsdienste in Anspruch nehmen zu können?	☐	☐	☐	☐	☐
2.25	Wie zufrieden sind Sie mit den Beförderungsmitteln, die Ihnen zur Verfügung stehen?	☐	☐	☐	☐	☐

In der folgenden Frage geht es darum, wie oft sich während der vergangenen zwei Wochen bei Ihnen negative Gefühle eingestellt haben, wie zum Beispiel Angst oder Traurigkeit.

		niemals	nicht oft	zeitweilig	oftmals	immer
2.26	Wie häufig haben Sie negative Gefühle wie Traurigkeit, Verzweiflung, Angst oder Depression?	☐	☐	☐	☐	☐

Teil 3: Anamnese

Der nachfolgende Fragebogen soll ihre gesundheitlichen Voraussetzungen zur Ausführung von körperlicher Tätigkeit einschätzen.

		ja	nein
3.1	Hat ihnen jemals ein Arzt gesagt, sie hätten “etwas am Herzen“ und ihnen Bewegung und Sport nur unter ärztlicher Kontrolle empfohlen?	☐	☐
3.2	Hatten Sie im letzten Monat Schmerzen in der Brust in Ruhe oder bei körperlicher Belastung (Anstrengung)?	☐	☐
3.3	Haben Sie Probleme mit der Atmung in Ruhe oder bei körperlicher Belastung?	☐	☐
3.4	Sind Sie jemals wegen Schwindel gestürzt oder haben Sie schon jemals das Bewusstsein verloren?	☐	☐
3.5	Haben Sie Knochen- oder Gelenkprobleme, die sich unter körperlicher Belastung verschlechtern könnten?	☐	☐
3.6	Hat Ihnen jemals ein Arzt ein Medikament gegen hohen Blutdruck oder wegen eines Herzproblems oder Atemproblems verschrieben?	☐	☐
3.7	Kennen Sie irgendeinen weiteren Grund, warum Sie nicht körperlich/ sportlich aktiv sein sollten?<br><br>--		

Teil 4: Befindlichkeit, Beschwerdeempfinden, Physis VOR

Dies ist eine Liste von Wörtern, mit denen man beschreiben kann, wie man sich augenblicklich fühlt. Bitte gehen Sie die Wörter der Liste nacheinander durch und entscheiden Sie sofort bei jedem Wort, wie stark es für Ihr augenblickliches Befinden zutrifft.
Beurteilen Sie nur, wie Sie sich **augenblicklich** fühlen. Geben Sie die Antwort, die Ihnen **unmittelbar** in den Sinn kommt. Wenn Ihnen eine Antwort schwer fällt, nennen Sie die, die am **ehesten** zutrifft. Bitte kreuzen Sie bei **jedem** Wort eine Antwort an und lassen Sie keines der Wörter aus.

Im Moment fühle ich mich…		gar nicht	kaum	Mitte	ziemlich	sehr
4.1	gedrückt	☐	☐	☐	☐	☐
4.2	ruhelos	☐	☐	☐	☐	☐
4.3	unbeschwert	☐	☐	☐	☐	☐
4.4	betrübt/bekümmert	☐	☐	☐	☐	☐
4.5	nachdenklich	☐	☐	☐	☐	☐
4.6	frisch	☐	☐	☐	☐	☐
4.7	passiv	☐	☐	☐	☐	☐
4.8	missmutig	☐	☐	☐	☐	☐
4.9	traurig	☐	☐	☐	☐	☐
4.10	beschaulich	☐	☐	☐	☐	☐
4.11	ärgerlich	☐	☐	☐	☐	☐
4.12	nach innen gekehrt	☐	☐	☐	☐	☐
4.13	angeregt	☐	☐	☐	☐	☐
4.14	locker	☐	☐	☐	☐	☐
4.15	nervös	☐	☐	☐	☐	☐
4.16	niedergeschlagen	☐	☐	☐	☐	☐
4.17	gelöst	☐	☐	☐	☐	☐
4.18	träumerisch	☐	☐	☐	☐	☐
4.19	angenehm/wohl	☐	☐	☐	☐	☐
4.20	verkrampft	☐	☐	☐	☐	☐
Im Moment fühle ich mich…		gar nicht	kaum	Mitte	ziemlich	sehr
4.21	energielos	☐	☐	☐	☐	☐
4.22	lasch	☐	☐	☐	☐	☐
4.23	unglücklich	☐	☐	☐	☐	☐
4.24	sauer	☐	☐	☐	☐	☐
4.25	träge	☐	☐	☐	☐	☐
4.26	angespannt	☐	☐	☐	☐	☐
4.27	gereizt/überreizt	☐	☐	☐	☐	☐
4.28	ausgezeichnet	☐	☐	☐	☐	☐
4.29	entspannt	☐	☐	☐	☐	☐
4.30	voller Energie	☐	☐	☐	☐	☐
4.31	besinnlich	☐	☐	☐	☐	☐
4.32	ruhig	☐	☐	☐	☐	☐
4.33	tatkräftig	☐	☐	☐	☐	☐
4.34	aktiv	☐	☐	☐	☐	☐
4.35	kribbelig/zappelig	☐	☐	☐	☐	☐
4.36	gut gelaunt	☐	☐	☐	☐	☐
4.37	mürrisch	☐	☐	☐	☐	☐
4.38	gelassen	☐	☐	☐	☐	☐
4.39	freudig/froh	☐	☐	☐	☐	☐
4.40	lahm	☐	☐	☐	☐	☐
Im Moment fühle ich mich…		gar nicht	kaum	Mitte	ziemlich	sehr
4.41	geborgen	☐	☐	☐	☐	☐
4.42	aufgeregt	☐	☐	☐	☐	☐
4.43	besorgt, dass etwas schief gehen könnte	☐	☐	☐	☐	☐
4.44	ausgeruht	☐	☐	☐	☐	☐
4.45	beunruhigt	☐	☐	☐	☐	☐
4.46	selbstsicher	☐	☐	☐	☐	☐
4.47	zufrieden	☐	☐	☐	☐	☐
4.48	besorgt	☐	☐	☐	☐	☐
4.49	vergnügt	☐	☐	☐	☐	☐

Es kommt häufig vor, dass sich die Stimmungslage verändert, während man sich bewegt. Manche Menschen empfinden Bewegung als angenehm, andere als unangenehm. Die Stimmungslage kann sich während der Bewegungszeit aber auch mehrfach verändern. Mit dieser Skala soll Ihre Stimmung **vor** dieser Bewegungseinheit erfasst werden. Bitte schätzen Sie Ihre Stimmungslage ein, indem Sie die entsprechende Zahl ankreuzen.

	Sehr gut		Gut		Ziemlich gut	Neutral	Ziemlich schlecht		Schlecht		Sehr schlecht
4.50	5	4	3	2	1	0	-1	-2	-3	-4	-5

Mit dieser Skala soll das Ausmaß an Aktivierung **vor** der Bewegungseinheit erhoben werden. Bitte kreuzen Sie jene Zahl an, die dem Ausmaß Ihrer momentanen Aktivierung am ehesten entspricht. Hohe Aktivierung könne Sie z.B. als Aufregung, Angst oder Ärger fühlen. Niedrige Aktivierung wird eher in Form von Gelassenheit, Entspannung oder Langeweile wahrgenommen.

	Niedrige Aktivierung					Hohe Aktivierung
4.51	1	2	3	4	5	6

Mit diesen Skalen soll Ihr subjektives Beschwerdeempfinden erhoben werden. Kreuzen Sie bitte auf der Linie ihr persönliches Befinden an. Die Endpunkte markieren hierbei jeweils die Extreme (z.B. „Kopfschmerzen: sehr“ = am stärksten vorstellbare Kopfschmerzen).

4.52	Wie würden Sie **im Moment** ihren Gesundheitszustand allgemein beurteilen?	☺ 😐 ☹<br>Sehr gut ——————— sehr schlecht
4.53	Wie stark gestresst fühlen Sie sich **im Moment**?	☺ 😐 ☹<br>Gar nicht ——————— sehr

Wie sehr litten Sie **innerhalb der letzten Stunde** unter…		
4.54	Kopfschmerzen	☺ 😐 ☹<br>Gar nicht ——————— sehr
4.55	Gelenksschmerzen (z.B. Kniegelenk, Hüftgelenk)	☺ 😐 ☹<br>———————
4.56	Herz- oder Brustschmerzen	☺ 😐 ☹<br>———————
4.57	Schwierigkeiten beim Atmen	☺ 😐 ☹<br>———————
4.58	Rückenschmerzen (Lenden-, Brust-, Halswirbelsäule)	☺ 😐 ☹<br>———————
4.59	Verdauungs-beschwerden (z.B. Übelkeit, Magenverstimmung)	☺ 😐 ☹<br>———————

Bitte beschreiben Sie auf der nachstehenden Skala Ihr **momentanes** Anstrengungsempfinden.

4.60 Borgskala: ______

Borgskala

6	überhaupt keine Anstrengung
7	extrem locker
8	
9	sehr locker
10	
11	locker
12	
13	ein wenig hart
14	
15	Hart
16	
17	Sehr hart
18	
19	extrem hart
20	maximale Anstrengung

© Gunnar Borg 1985

4.61 Blutdruck: sys:______ dia:______ mmHg
4.62 Puls (ok?) ☐
4.63 Herzratenvariabilität (ok?) ☐
4.64 Speichelprobe (ok?) ☐
4.65 Kapillarblut (ok?) ☐

Teil 5: Befindlichkeit, Beschwerdeempfinden, Physis ZWISCHEN

Es werden erneut die gleichen Items wie in Teil 4 erhoben.

Teil 6: Befindlichkeit, Beschwerdeempfinden, Physis NACH

Es werden erneut die gleichen Items wie in Teil 4 erhoben.
Teil 6 beinhaltet zusätzlich noch folgendes Item:

6.61 Wie beeinflusste die Umgebung, in der Sie sich eben befunden haben, ihr Wohlbefinden?

1	2	3	4	5	6	7	8	9	10

100% positiv — 50% positiv 50% negativ — 100% negativ

Teil 1, 2 und 3 wird einmalig zur ersten Bedingung erhoben.
Teil 5 und 6 ist eine Wiederholung von Teil 4 nach dem Aufstieg bzw. Abstieg.
Teil 4, 5 und 6 müssen zur zweiten und dritten Bedingung erneut ausgefüllt werden.

D) Case Report Form B

Case Report Form B unterschiedet sich von Case Report Form A folgendermaßen:

- Teil 3: Anamnese entfällt in Teilprojekt B.
- Die physiologischen Parameter werden nicht erhoben (*4.61-4.65, 5.61-5.65, 6.62-6.66*).
- Die Probanden werden nur 2 Bedingungen ausgesetzt.
- Der letzte Teil umfasst zudem folgendes Item:

Wie schätzen Sie insgesamt die Naturbelassenheit der Umgebung ein, in der Sie sich eben befunden haben?

1	2	3	4	5	6	7	8	9	10

100% künstlich

50% naturbelassen
50% künstlich

100% naturbelassen

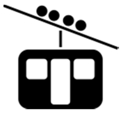

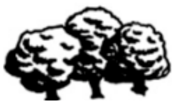

E) Case Report Form C

Die Fragebögen zu Teilprojekt C (International Physical Activity Questionnaire (IPAQ), Physical Activity Questionnaire (PAQ), Work Ability Index (WAI), Kessler K-10 (K10), Quality of Life Comic (QOLC)) sind unter folgendem Link ersichtlich: http://www.fragebogen.pmu.ac.at/index.php?r=survey/index/sid/541753/lang/de.

F) Versicherungspolizze

Wird nachgereicht.

G) Verwendete Literatur

Bourdeau, P., J. Corneloup and P. Mao (2002). "Adventure sports and tourism in the French Mountains: dynamics of change and challenges for sustainable development." Current Issues in Tourism 5(1): 22-32.

Buckworth, J. (2013). Exercise psychology. Champaign, Ill. [u.a.], Champaign, Ill. [u.a.]: Human Kinetics.

Burtscher, M. (1999). "Höhenkopfschmerz: Epidemiologie, Pathophysiologie, Therapie und Prophylaxe. [High altitude headache: epidemiology, pathophysiology, therapy and prophylaxis]." Wiener Klinische Wochenschrift 111(20): 830-836.

Deslandes, A. C. (2014). "Exercise and Mental Health: What did We Learn in the Last 20 Years?" Front Psychiatry 5: 66.

Hamer, M., A. Taylor, and A. Steptoe. (2006). "The effect of acute aerobic exercise on stress related blood pressure responses: a systematic review and meta-analysis." Biological Psychology 71 (2):183-90.

Kuratorium für Verkehrssicherheit. (2013). "Freizeitunfallstatistik." Retrieved 11.02.2015, 2015, from http://www.kfv.at/.

Lees, C. and J. Hopkins (2013). "Effect of aerobic exercise on cognition, academic achievement, and psychosocial function in children: a systematic review of randomized control trials." Preventing Chronic Disease 10: 174.

North, T. C., P. McCullagh and Z. V. Tran (1990). "Effect of Exercise on Depression." Exercise and Sport Sciences Reviews 18(1): 379-416.

Paffenbarger, R. S., Jr., A. L. Wing and R. T. Hyde (1978). "Physical activity as an index of heart attack risk in college alumni." American Journal of Epidemiology 108(3): 161-175.

Penedo, F. J. and J. R. Dahn (2005). "Exercise and well-being: a review of mental and physical health benefits associated with physical activity." Current opinion in psychiatry 18(2): 189–193.

Petruzzello, S., D. Landers, B. Hatfield, K. Kubitz and W. Salazar (1991). "A Meta-Analysis on the Anxiety-Reducing Effects of Acute and Chronic Exercise." Sports Medicine 11(3): 143-182.

Pretty, J., J. Peacock, M. Sellens, and M. Griffin. 2005. "The mental and physical health outcomes of green exercise." International Journal of Environmental Health Research 15 (5):319-337.

Reddy, K. S. (2004). "Cardiovascular disease in non-Western countries." New England Journal of Medicine: 2438-2510.

Sturm, J., M. Plöderl, C. Fartacek, K. Kralovec, D. Neunhäuserer, D. Niederseer, W. Hitzl, J. Niebauer, G. Schiepek and R. Fartacek (2012). "Physical exercise through mountain hiking in high-risk suicide patients. A randomized crossover trial." Acta Psychiatrica Scandinavica 126(6): 467-475.

Thompson Coon, J., K. Boddy, K. Stein, R. Whear, J. Barton and M. H. Depledge (2011). "Does Participating in Physical Activity in Outdoor Natural Environments Have a Greater Effect on Physical and Mental Wellbeing than Physical Activity Indoors? A Systematic Review." Environmental Science & Technology 45(5): 1761-1772.

Yeung, R. R. (1996). "The acute effects of exercise on mood state." Journal of Psychosomatic Research 40(2): 123-141.

Beirat für ethische Fragen in der wissenschaftlichen Forschung, Universität Innsbruck
(Geschäftsstelle: Büro der Vizerektorin für Forschung
Homepage: http://www.uibk.ac.at/rektorenteam/forschung/ethikbeirat.html)

INSTITUT FÜR SPORTWISSENSCHAFT DER UNIVERSITÄT INNSBRUCK

Fürstenweg 185
6020 Innsbruck

universität innsbruck

Arbeitsbereich Sportpsychologie:

Leiter Univ.-Prof. Dr. Martin Kopp

Forschungsprojekt

Effekte des Bergsports auf die individuelle Lebensqualität und Gesundheit

Univ.-Prof. Dr. Martin Kopp

Institut für Sportwissenschaft

HIER

An das

Review Board für sportwissenschaftliche Forschungsprojekte

Univ.-Prof. Dr. Elmar Kornexl

Institut für Sportwissenschaft

Universität Innsbruck

Fürstenweg 185

A-6020 Innsbruck

Innsbruck, den 22.04.2015

Antrag auf Begutachtung eines Forschungsprojektes

Sehr geehrte Damen und Herren,

ich bitte um Begutachtung des geplanten Forschungsprojektes „Effekte des Bergsports auf die individuelle Lebensqualität und Gesundheit" im Review Board.

Mit freundlichen Grüßen,

Martin Kopp

Anlagen:
- Projektbeschreibung (1)
- Projektgruppe (4)
- Aufstellung Drittmittel/Projektkosten (s.o.)
- Probandenversicherung (s.o.)
- Probandeninformation und Einwilligungserklärung (s.o.)

1. Einleitung und aktueller Forschungsstand

Auswirkungen von körperlicher Aktivität auf die physische und psychische Gesundheit werden in der Literatur eingehend beschrieben (1-5). Bisher vorliegende Interventionsstudien haben meist die Wirkung von aerobem Ausdauersport ohne die Überwindung von Höhenunterschieden (z.B. Radfahren, Wandern in der Ebene, Spielsportarten) untersucht (6). Sowohl auf die physische als auch auf die psychische Gesundheit konnten positive Effekte gezeigt werden. So ist beispielsweise eine erhöhte körperliche Aktivität mit einem reduzierten Risiko koronare Herzkrankheiten (1), degenerative Leiden und Infektionskrankheiten zu entwickeln (7) assoziiert Außerdem reduziert körperliche Aktivität Angst (8) und Depression (9) und zeigt positive Effekte auf die Befindlichkeit (6).

Neben den oben genannten Sportarten spielt bei Menschen aus Alpenländern der Bergsport eine zentrale Rolle (10). Der Umstand, dass sich die Berge direkt vor der Haustüre befinden, lädt viele Menschen dazu ein, Sportarten wie beispielsweise Bergwandern, Hochtouren gehen, Skitourengehen, Mountainbiking und weitere auszuüben. Die Anziehungskraft der Berge beschränkt sich jedoch nicht nur auf Menschen aus Ländern mit Bergregionen. Neben dem Meer sind die Berge eine beliebte Urlaubsdestination. Allein in den Alpen werden jährlich 40 Millionen Bergtouristen gezählt (11).

Die physische Aktivität beim Bergsport unterscheidet sich jedoch von der physischen Aktivität ohne die Überwindung von Höhenunterschieden hinsichtlich der äußeren (z.B. Temperaturveränderung) und inneren Faktoren (z.B. Art der Muskelarbeit). Es stellt sich also die Frage, ob der Bergsport vergleichbare Effekte auf individuelle Lebensqualität und Gesundheit zeigt. Bisher wurde zu dieser Thematik nur eine Pilotstudie an Suizidpatienten durchgeführt (12). Zu gesunden Probanden sind noch keine Daten vorliegend.

2. Zielsetzung

Ziel der vorliegenden Arbeit ist, es herauszufinden, welche unmittelbaren und chronischen Auswirkungen der Bergsport auf die individuelle Lebensqualität, die psychische Gesundheit und die körperliche Beschwerdewahrnehmung hat.

3. Methode

Das Forschungsprojekt beinhaltet 3 Teilprojekte A, B und C. Potentielle akute Effekte werden in Teilprojekt A und B untersucht, etwaige chronische Effekte werden in Teilprojekt C behandelt.

3.1 Probanden

Teilprojekt A: Untersucht werden 30 Personen im Alter von 18-60 Jahren, welche sich zur Teilnahme an der Studie bereit erklären. Die Probanden werden freiwillig im Raum Innsbruck mittels Emailausschreibung rekrutiert. Ausschlusskriterien sind akute Schwangerschaft, Stillperiode, bestehende oder im Verlauf der Untersuchungen diagnostizierte chronische oder akute Erkrankungen. Einschlusskriterien sind Alter zwischen 18 und 60 Jahren und Fähigkeit und Bereitschaft zu einer 3stündigen Bergwanderung.

Teilprojekt B: Untersucht werden etwa 60 Personen im Alter von 18-60 Jahren, welche sich zur Teilnahme an der Studie bereit erklären. Die Probanden werden freiwillig in Sektionsveranstaltungen des Österreichischen Alpenvereins rekrutiert. Ausschlusskriterien sind akute Schwangerschaft, Stillperiode, bestehende oder im Verlauf der Untersuchungen diagnostizierte chronische oder akute Erkrankungen. Einschlusskriterien sind Alter zwischen 18 und 60 Jahren und Teilnahme am Kursangebot des Österreichischen Alpenvereins.

Teilprojekt C: Untersucht werden etwa 2000 Personen mittels Online Fragebogen. Die Probanden werden mittels Emailaussendungen im Raum Österreich, Italien und Deutschland rekrutiert. Die Probanden nehmen freiwillig teil und können die Umfrage jederzeit verlassen, weshalb für dieses Teilprojekt keine Einverständniserklärung notwendig ist.

Für die Kalkulation der Stichprobengröße wurden Poweranalysen unter folgenden Annahmen durchgeführt:

Teilprojekt A: Nach akuter Bewegungsintervention berichten Hamer et al. (2006) (13) in ihrer Metaanalyse eine Gesamteffektstärke von d=0,40 für die Reduktion physiologischer Stressparameter. Dieser Effekt erhöht sich, wenn die Bewegung länger andauert (1h: d=0,99). Bei einer Bewegungsintervention von etwa 3 Stunden wird daher mit einem großen Effekt von d=1,3 gerechnet. Um einen Effekt von d=1,3 bei $\alpha=0,05$ und $1-\beta=0,8$ nachzuweisen, sind für drei Messzeitpunkte mindestens 25 Probanden notwendig. Wenn eine Dropout-Rate von 20% mit einbezogen wird, ergibt sich ein Mindestumfang von 30 Probanden. Um diese Kalkulation abzusichern wird eine Voruntersuchung durchgeführt.

Teilprojekt B: Thompson Coon et al. (2011) (14) beschreiben in ihrem Review über die Unterschiede bei körperlicher Aktivität indoor bzw. outdoor einen mindestens moderaten Effekt auf die Befindlichkeit zu Gunsten der Aktivität outdoor (d=0,42). Unterschiede zwischen angenehm und unangenehm wahrgenommener Umgebung outdoor untersuchten Pretty et al. (2005) (15). Die Effektstärken reichen je nach Subskala der Befindlichkeit von d=0,41 (Angst) bis d=1,75 (Depression). Um einen Effekt von d=0,41 bei $\alpha=0,05$ (zweiseitig) und $1-\beta=0,8$ nachzuweisen, sind mindestens 49 Probanden notwendig. Wenn eine Dropout-Rate von 20% mit einbezogen wird, ergibt sich ein Mindestumfang von 60 Probanden.

Teilprojekt C: Da es bei diesem Projekt um einen möglichst umfassenden Einblick in das Bewegungs- und Bergsportverhalten sowie die Lebensqualität von bergsportaktiven Personen und zudem diverse Subgruppenanalysen geplant sind, wurde keine Poweranalyse durchgeführt.

3.2 Verwendete Messverfahren

Für die Durchführung der Untersuchung werden folgende Fragbögen verwendet und folgende physiologische Messungen durchgeführt.

3.2.1 Physical Activity Readiness Questionnaire (PARQ) (16)

An Hand des PAR-Q Fragebogens bewerten die Probanden an Hand von 7 Fragen nach („ja"/"nein") subjektiv, inwiefern sie die gesundheitlichen Voraussetzungen zur Ausführung von körperlicher Tätigkeit besitzen. Sollten die Probanden mindestens eine der Fragen mit „ja" beantworten und dennoch bei der Studie teilnehmen wollen, werden die Probanden gebeten, ein ärztliches Attest des Hausarztes vorzuweisen.

3.2.2 Soziodemographische Daten (SD)

Durch die soziodemographischen Daten werden die einzelnen Merkmale der Probanden festgehalten. Anhand dieser Daten können die Versuchspersonen genau beschrieben und analysiert werden.

3.2.3 World Health Organization Quality of life Assessment (WHOQOL) (17)

Mittels 26 Items auf einer 5stufigen Likertskala wird die Lebensqualität in den letzten 2 Wochen subjektiv beschrieben. Diese Daten sollen einen Überblick über die allgemeine Lebenszufriedenheit geben und sind nicht auf die Intervention bezogen.

3.2.4 Befindlichkeitsskalen (BFS) (18), State Trait Anxiety Inventory (STAI) (19)

Mit der Erhebung aktueller Befindlichkeiten vor und unmittelbar nach der Ausübung von moderater körperlicher Aktivität sollen die Auswirkungen von Bewegungseinheiten auf den aktuellen momentanen psychophysischen Zustand einer Person erfasst werden. Im deutschsprachigen Raum kommen zumeist die von Abele/Brehm (1986) erstellten Befindlichkeitsskalen zur Verwendung. Unter Befindlichkeit ist nach Abele/Brehm der jeweils momentane Zustand eines Menschen zu verstehen. Die Abgrenzung zu den Emotionen ist durch die Betonung der Ungerichtetheit gegeben.

Diese Messmethode geht davon aus, dass sich Menschen an bereits erlebte Befindlichkeiten erinnern und diese bewerten können. Die Befindlichkeitsskalen bestehen aus einer Adjektivliste mit 40 Items (8 Subskalen) mit einem 5stufigen Antwortmodus.

Das State Trait Anxiety Inventory ist ein Instrument, das die Erfassung von sowohl habitueller als auch situativer Angst ermöglicht. Da es in der vorliegenden Studie um die Auswirkung von bestimmten Interventionen geht, wird nur die Subskala der situativen Angst verwendet. Diese besteht ähnlich wie die Befindlichkeitsskalen aus 20 Adjektiven, die von den Probanden in einem 4stufigen Modus bewertet werden müssen. Da sich die verwendeten Adjektive teilweise wörtlich mit den Befindlichkeitsskalen decken, wird das State Trait Anxiety Inventory dahin gehend modifiziert, dass ein 5stufiger Antwortmodus gewählt wird und die Adjektive, die bereits in den Befindlichkeitsskalen abgefragt wurden, nicht nochmals bewertet werden müssen.

3.2.5 Felt Arousal Scale (FAS) (20)

Die Felt Arousal Scale ist eine Single Item Erhebung zur Messung des erlebten Aktivierungs- bzw. Erregungsniveaus. Die bipolare Skala reicht von 1 (niedrige Erregung) bis 5 (hohe Erregung). Mit „Erregung“ wird gemeint, wie „aufgewühlt“ sich die Probanden fühlen. Sie können hohe Erregung in einer Vielzahl von Möglichkeiten erleben, zum Beispiel als Aufregung, Angst oder Wut. Niedrige Erregung wird hingegen als Entspannung, Langeweile oder Ruhe empfunden.

3.2.6 Feeling Scale (FS) (21)

Während der Ausführung der körperlichen Tätigkeit kann es zu einer Veränderung der Stimmung kommen. Einige der Teilnehmer empfinden die ausführende Tätigkeit als angenehm, während andere sie als unangenehm empfinden. Darüber hinaus kann das Gefühl über die Zeit schwanken. Folgende Schwankungen können einige Male während der ausführenden Tätigkeiten empfunden werden. Für die Messung solcher Schwankungsreaktionen haben die Wissenschaftler Hardy & Rejeski die vorliegende Single Item Skala mit 11stufigem Antwortmodus entworfen.

3.2.7 Visuelle Analogskala (VAS) (22)

Eine visuelle Analogskala besteht aus einer Frage oder Aussage und den 2 extremen Ausprägungen als Endpunkte. Zwischen den Extremen kann sich der Proband auf einer kontinuierlichen Skala einordnen. Eine visuelle Analogskala hat den Vorteil einer sehr kurzen Bearbeitungszeit und mit anderen Skalen vergleichbaren Validierungskennwerten (22).

In der vorliegenden Studie werden visuelle Analogskalen für die körperliche Beschwerdewahrnehmung und das subjektive Stressempfinden verwendet.

3.2.8 Rating of Perceived Exertion (RPE) (23)

Mit der Rating of Perceived Exertion (auch Borg Skala) kann der Leistungsaufwand, die Erschöpfung und Ermüdung, sowie das Anstrengungsempfinden während körperlicher Belastung erhoben werden. Die Borg Skala berücksichtigt den Fitness-Level. Sie bewertet die körperliche Arbeit innerhalb einer Skala zwischen 6 (kein Gefühl von Anstrengung) und 20 (sehr, sehr hart). Moderate Aktivitäten fallen in den Bereich 11 bis 14 auf der Borg Skala („ziemlich leicht" bis „etwas schwer"), während sportliche Aktivitäten in der Regel in den Bereich der Nummern 15 und höher („hart" bis „sehr, sehr hart") eingestuft werden (23).

3.2.9 International Physical Activity Questionnaire (IPAQ) (24)

Dieser viel verwendete Fragebogen versucht zu erfassen, wie hoch die körperliche Aktivität im täglichen Leben der Probanden ist. In insgesamt 7 Items werden verschiedene Intensitäten von körperlicher Aktivität abgefragt, die mit ja oder nein beantwortet werden. Bei positiver Antwort müssen die verschiedenen Intensitäten mit Tagen, Stunden und Minuten quantifiziert werden. Als Bemessungsgrundlage werden die letzten 7 Tage verwendet. Dieser Fragebogen erlaubt die Abschätzung der körperlichen Aktivität in der Einheit des metabolischen Äquivalentes (MET).

3.2.10 Physical Activity Questionnaire (PAQ) (25)

Ein 2 Items Fragebogen, der den Grad der körperlichen Aktivität bestimmt: „Beschreiben Sie Ihre körperliche Bewegung bei der Arbeit/in der Freizeit". Die Items werden in einem 4 bzw. 5stufigen Modus beantwortet.

3.2.11 Work Ability Index (WAI) (26)

Aus dem umfangreichen Fragebogen zur Arbeitsfähigkeit wird nur die Frage nach den Grunderkrankungen verwendet. Dabei werden 13 Beispiele von Grunderkrankungen aufgezählt und in einem 3stufigen Antwortmodus („eigene Diagnose", „Diagnose vom Arzt", „liegt nicht vor") beantwortet.

3.2.12 Kessler K-10 (K10) (27)

Der Kessler K-10 erfasst in 10 Items und einem 5stufigen Antwortmodus den unspezifischen psychsichen Distress der letzten 30 Tage. Es wird erfragt, wie häufig sich die Probanden bespielsweise „nervös“ gefühlt haben.

3.2.13 Quality of Life Comic (QOLC)

Der Quality of Life Comic wurde von der Paracelsus Universität in Salzburg entwickelt und stellt 7 Fragen nach der Lebensqualität, die mittels einer Art visuellen Analogskala, deren Skalen durch entsprechende Bilder hinterlegt sind, beantwortet werden.

3.2.14 Physiologische Messungen

In Absprache mit dem Leiter des Labors für Translationale Immunforschung der Paracelsus Medizinische Privatuniversität Salzburg Univ. Doz. Dr. Arnulf Josef Hartl werden zudem Speichel- oder Kapillarblutproben erhoben und immunologische Faktoren und Stressparameter festgestellt. Daneben wird die Herzfrequenz, Herzratenvariabilität und der Blutdruck gemessen.

3.3 Untersuchungsdesign

Teilprojekt A besteht für alle Teilnehmenden neben einer Anamnese aus 3 Bedingungen, die randomisiert zugeteilt werden und in einem Gesamtzeitraum von 3 Wochen stattfinden: Bergwanderung, Laufbandeinheit (je moderate Intensität), Kontrollsituation. Vor, während und nach den Bedingungen werden Fragebogenerhebungen (Lebensqualität, Befindlichkeit, Stress, Depression, Angst, körperliches Beschwerdeempfinden) sowie physiologische Messungen (Speichel-, Kapillarblutproben, Herzfrequenz, Blutdruck) durchgeführt (*randomized within-subject design*).
Neben der Beantwortung der Frage nach möglichen Unterschieden zwischen einer Kontroll- und Bergsportsituation sollen durch die Laufbandeinheit auch potentielle Effekte isoliert werden, die ausschließlich auf die Bewegung bzw. das Naturerlebnis zurück zu führen sind.

In Teilprojekt B werden andere Probanden als in Teilprojekt A untersucht. Es besteht für alle Teilnehmenden aus 2 Bedingungen, die randomisiert zugeteilt werden: Bergwanderung in Räumen mit großem anthropogenem Einfluss, Bergwanderung in Räumen mit geringem anthropogenen Einfluss (je moderate Intensität). Vor, während und nach den Bedingungen werden die gleichen Fragebogenerhebungen wie in Teilprojekt A durchgeführt. Es werden keine physiologischen Parameter erhoben (*randomized within-subject design*).
Damit soll vor allem der Frage nachgegangen werden, ob sich Bewegung in der Natur mit großem anthropogenem Einfluss anders als in weitgehend unberührter Natur auswirkt.

Teilprojekt C ist eine Online-Fragebogenerhebung. Es werden Bergaktive mit der österreichischen Bevölkerung verglichen und Unterschieds- und Korrelationsanalysen durchgeführt um die Frage nach potentiellen Langzeiteffekten von Bergsport zu beantworten. Dazu wird das Bewegungsverhalten, die Lebensqualität, die Befindlichkeit und Arbeitsfähigkeit der Probanden erfasst (*between-subject design*).

3.3.1 Teilprojekt A

Vor Beginn der eigentlichen Untersuchung erfolgt die Information, Anamnese und Einverständniserklärung der Probanden. Bei Erfüllen der Einschlusskriterien werden soziodemographische Daten, allgemeiner Gesundheitszustand und Lebensqualität erfasst. Im Anschluss werden die Probanden randomisiert zu 3 verschiedenen Bedingungen zugeteilt, die alle Probanden durchlaufen. Jeweils vor, während und nach den Bedingungen werden Fragebogenerhebungen und physiologische Messungen durchgeführt.
Bergwanderbedingung:

Die Probanden nehmen an einer vom Projektkoordinator geführten Bergwanderung teil. Die Gesamtdauer beträgt 3 Stunden und beinhaltet Aufstieg, Messungen, Pausen und Abstieg. Dabei werden ca. 700 Höhenmeter überwunden. Die Intensität wird moderat gewählt (RPE: 11-14).

Laufbandbedingung:

Die Laufbandbedingung wird der Bergwanderbedingung so gut wie möglich bzgl. der Dauer, Steigung, Intensität und Pausengestaltung angepasst.

Kontrollsituation:

In der Kontrollsituation werden die Probanden angewiesen eine sitzende Position einzunehmen. Die Probanden können dabei lesen, fernsehen, am PC arbeiten o.ä. mit der Vorgabe, sich nicht anzustrengen (RPE: 6). Die Dauer beträgt ebenfalls 3 Stunden.

Für einen genauen Überblick über den zeitlichen Ablauf der Fragebögen und Messungen, vgl. 5.1.1.

3.3.2 Teilprojekt B

Wie in Teilprojekt A erfolgt vor Beginn der eigentlichen Untersuchung die Information, Anamnese und Einverständniserklärung der Probanden. Bei Erfüllen der Einschlusskriterien werden soziodemographische Daten, allgemeiner Gesundheitszustand und Lebensqualität erfasst. Im Anschluss werden die Probanden randomisiert zu 2 verschiedenen Bedingungen zugeteilt, die alle Probanden durchlaufen. Jeweils vor, während und nach den Bedingungen werden Fragebogenerhebungen, jedoch keine physiologische Messungen durchgeführt. Die Bergsportbedingungen finden im Rahmen von Sektionsveranstaltungen des Österreichischen Alpenvereins statt, wobei die Tourenführer vom Koordinationsleiter instruiert werden. Der Bergsport beschränkt sich nicht nur auf Bergwandern, sondern auf verschiedene Spielarten des Bergsports.

Bergsportbedingung in Räumen mit großem anthropogenem Einfluss:

Die Sektionsveranstaltung wird in einem Raum mit großem anthropogenem Einfluss durchgeführt (Lifte, Skipisten, Häuser etc.).

Bergsportbedingung in Räumen mit geringem anthropogenem Einfluss:

Die Sektionsveranstaltung wird in der so wenig wie möglich vom Menschen berührten Natur durchgeführt.

Für einen genauen Überblick über den zeitlichen Ablauf der Fragebögen, vgl. 5.1.2.

3.3.3 Teilprojekt C

Es wird eine Online Fragebogenerhebung durchgeführt. Über verschiedene Informationskanäle der Projektpartner wird versucht, möglichst viele Teilnehmer im Alpenraum zu erreichen.
Für einen genauen Überblick über die verwendeten Fragebögen, vgl. 5.1.3.

3.4 Statistische Auswertung

Für Teilprojekt A und B werden für die Bearbeitung der Frage nach potentiellen Unterschieden in den Messzeitpunkten und Bedingungen je nach Skalenniveau und Verteilung Varianzanalysen mit Messwiederholung (Faktoren: Messzeitpunkt 3fach, Bedingung 2- bzw. 3fach) oder Friedmann-Tests gewählt. Für Subgruppenanalysen werden ausgewählte Variablen als Gruppenfaktoren verwendet.
Bei Teilprojekt C werden Prüfverfahren zu Unterschieden und Korrelationen angewendet. Je nach Skalenniveau und Subgruppenanzahl kommen dazu Varianzanalysen, Kruskal-Wallis-Test, t-Test, Mann-Whitney-U-Test sowie Spearman-Rangkorrelation und Pearson-Maßkorrelation in Betracht.
Das Signifikanzniveau wird mit 5% festgelegt.

4. Projektgruppe

Österreichischer Alpenverein (OeAV) vertreten durch: Mag. Michael Larcher (Leiter Abteilung Bergsport), Dipl. Sozialpäd. (FH) Jürgen Einwanger (Projektleiter „Bergsport&Gesundheit" beim OeAV)

Paracelsus Medizinische Privatuniversität Salzburg (PMU) - Institut für Physiologie und Pathophysiologie vertreten durch: Univ. Doz. Dr. Arnulf Josef Hartl (Leiter des Labors für Translationale Immunforschung)

Leopold-Franzens-Universität Innsbruck (LFU) - Institut für Sportwissenschaft vertreten durch: Univ.-Prof. Dr. Martin Kopp (Projektleiter LFU), Martin Niedermeier (PhD Student, Projektkoordination)

5. Anhang

5.1 Untersuchungsdesign

5.1.1 Teilprojekt A

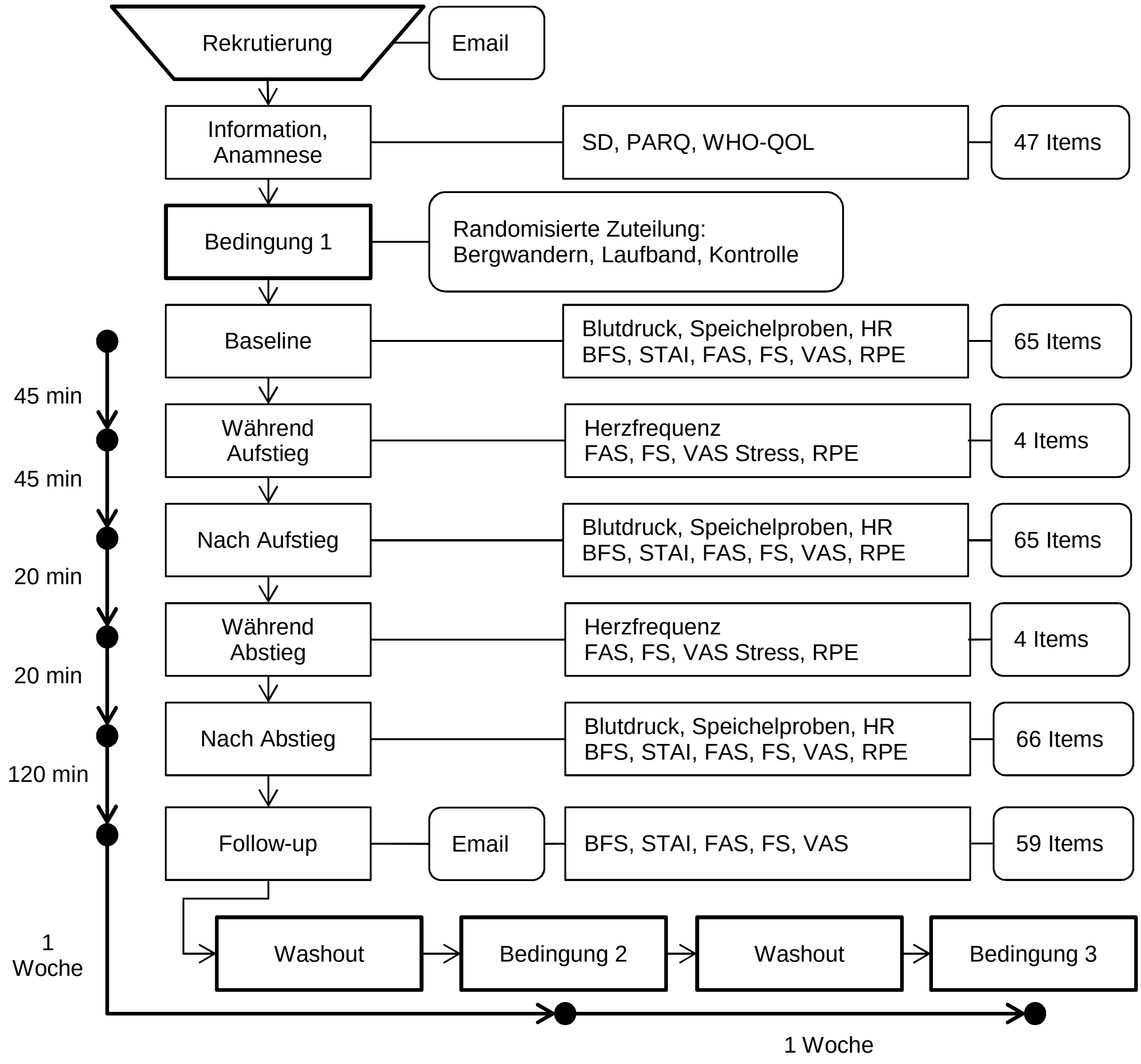

Gesamt: 19 Messzeitpunkte, 836 Items pro Proband.

5.1.2 Teilprojekt B

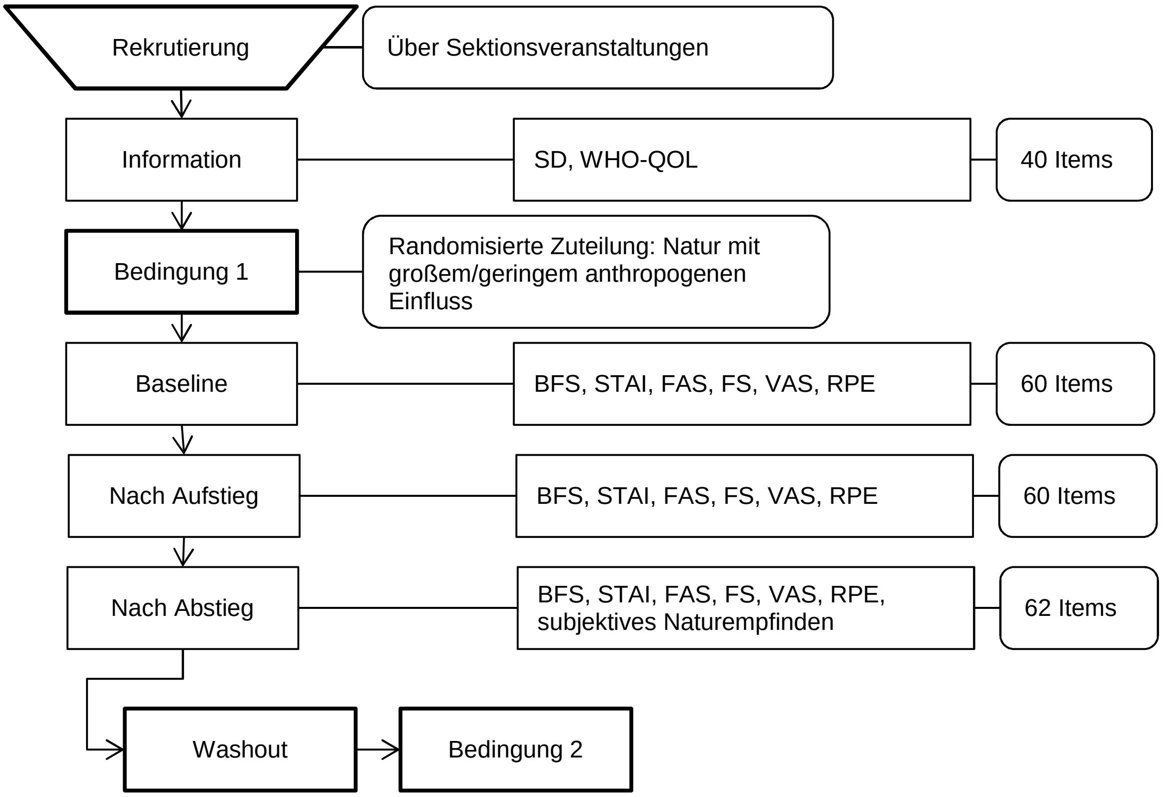

Gesamt: 7 Messzeitpunkte, 404 Items pro Proband.

5.1.3 Teilprojekt C

SD1
- Soziodemographische Daten1
- Geschlecht, Alter, Größe, Gewicht, Postleitzahl

EQ5D
- Beweglichkeit, für sich selbst sorgen, Alltag, Schmerzen, Angst/Niedergeschlagenheit
- Gesundheitszustand

PAQ
- Physical Activity Questionnaire
- Körperliche Aktivität Arbeit/Freizeit

BS
- Bergsport
- Mitgliedschaft alpiner Verein, hauptsächlich ausgeübte Bergsportart

IPAQ
- International Physical Activity Questionnaire
- anstrengende/moderate Aktivitäten, Fußweg, Sitzen

FS
- Feeling Scale
- indoor/outdoor

K10
- Kessler 10

WAI
- Work Ability Index
- Grunderkrankungen

SD2
- Soziodemograpfische Daten2
- Familienstand, Bildung, Beruf, Einkommen, Migrationshintergrund, Geburtsland

QOLC
- Quality of Life Comic

Gesamt: 1 Messzeitpunkt, 63 Items pro Proband.

6. Literaturverzeichnis

1. Paffenbarger RS, Jr., Wing AL, Hyde RT. Physical activity as an index of heart attack risk in college alumni. Am J Epidemiol. 1978;108(3):161-175.
2. Reddy KS. Cardiovascular disease in non-Western countries. N Engl J Med. 2004:2438-2510.
3. Deslandes AC. Exercise and Mental Health: What did We Learn in the Last 20 Years? Front Psychiatry. 2014;5:66.
4. Lees C, Hopkins J. Effect of aerobic exercise on cognition, academic achievement, and psychosocial function in children: a systematic review of randomized control trials. Prev Chronic Dis. 2013;10:174.
5. Penedo FJ, Dahn JR. Exercise and well-being: a review of mental and physical health benefits associated with physical activity. Current opinion in psychiatry. 2005;18(2):189–193.
6. Yeung RR. The acute effects of exercise on mood state. J Psychosom Res. 1996;40(2):123-141.
7. Schwarzer R. Psychologie des Gesundheitsverhaltens. 2. Auflage. Göttingen (u.a.): Hogrefe Verlag; 1996.
8. Petruzzello S, Landers D, Hatfield B, Kubitz K, Salazar W. A Meta-Analysis on the Anxiety-Reducing Effects of Acute and Chronic Exercise. Sports Med. 1991;11(3):143-182.
9. North TC, McCullagh P, Tran ZV. Effect of Exercise on Depression. Exerc Sport Sci Rev. 1990;18(1):379-416.
10. Bourdeau P, Corneloup J, Mao P. Adventure sports and tourism in the French Mountains: dynamics of change and challenges for sustainable development. Current Issues in Tourism. 2002;5(1):22-32.
11. Burtscher M. Höhenkopfschmerz: Epidemiologie, Pathophysiologie, Therapie und Prophylaxe. [High altitude headache: epidemiology, pathophysiology, therapy and prophylaxis]. Wien Klin Wochenschr. 1999;111(20):830-836.
12. Sturm J, Plöderl M, Fartacek C, Kralovec K, Neunhäuserer D, Niederseer D, et al. Physical exercise through mountain hiking in high-risk suicide patients. A randomized crossover trial. Acta Psychiatr Scand. 2012;126(6):467-475.
13. Hamer M, Taylor A, Steptoe A. The effect of acute aerobic exercise on stress related blood pressure responses: a systematic review and meta-analysis. Biol Psychol. 2006;71(2):183-190.
14. Thompson Coon J, Boddy K, Stein K, Whear R, Barton J, Depledge MH. Does Participating in Physical Activity in Outdoor Natural Environments Have a Greater Effect on Physical and Mental Wellbeing than Physical Activity Indoors? A Systematic Review. Environ Sci Technol. 2011;45(5):1761-1772.
15. Pretty J, Peacock J, Sellens M, Griffin M. The mental and physical health outcomes of green exercise. Int J Environ Health Res. 2005;15(5):319-337.
16. Shephard R. Readiness for physical activity. President's Council on Physical Fitness and Sports Research Digest; 1994.
17. Organization WH. Development of the World Health Organization WHOQOL-BREF Quality of Life Assessment. Psychol Med. 1998;28(03):551-558.
18. Abele-Brehm A, Brehm W. Zur Konzeptualisierung und Messung von Befindlichkeit: Die Entwicklung der "Befindlichkeitsskalen" (BFS). [The conceptualization and measurement of mood: The development of the "Mood Survey."]. Diagnostica. 1986;32:209-228.
19. Spielberger CD, Gorsuch RL, Lushene RE. Manual for the state-trait anxiety inventory. Palo Alto, CA: Consulting Psychologists Press; 1970.
20. Svebak S, Murgatroyd S. Metamotivational dominance: A multimethod validation of reversal theory constructs. J Pers Soc Psychol. 1985;48(1):107.
21. Hardy CJ, Rejeski WJ. Not what, but how one feels: The measurement of affect during exercise. J Sport Exerc Psychol. 1989;11(3).
22. Lesage FX, Berjot S, Deschamps F. Clinical stress assessment using a visual analogue scale. Occup Med (Lond). 2012;62(8):600-605.

23. Borg G. Psychophysical bases of perceived exertion. Med Sci Sports Exerc. 1982(14):377–381.

24. Booth ML, Ainsworth BE, Pratt M, Ekelund U, Yngve A, Sallis JF, et al. International physical activity questionnaire: 12-country reliability and validity. Med Sci Sports Exerc. 2003;195(9131/03):3508-1381.

25. Johansson G, Westerterp K. Assessment of the physical activity level with two questions: validation with doubly labeled water. Int J Obes (Lond). 2008;32(6):1031-1033.

26. Tuomi K, Ilmarinen J, Jahkola A, Katajarinne L, Tulkki A. Work Ability Index. Helsinki: Finnish Institute of Occupational Health; 1998.

27. Kessler RC, Andrews G, Colpe LJ, Hiripi E, Mroczek DK, Normand S-L, et al. Short screening scales to monitor population prevalences and trends in non-specific psychological distress. Psychol Med. 2002;32(06):959-976.
